# Characterization of the Intrinsic Phospholipase A1 Activity of *Bordetella pertussis* Adenylate Cyclase Toxin

**DOI:** 10.3390/toxins10120514

**Published:** 2018-12-04

**Authors:** David González-Bullón, César Martín, Helena Ostolaza

**Affiliations:** Department of Biochemistry and Molecular Biology, Biofisika Institute (UPV/EHU, CSIC), University of Basque Country, Aptdo. 644, 48080 Bilbao, Spain; david_go88@hotmail.com (D.G.-B.); cesar.martin@ehu.eus (C.M.)

**Keywords:** bacterial toxin, *Bordetella pertussis*, adenylate cyclase toxin, phospholipase A activity

## Abstract

Adenylate cyclase toxin (ACT, CyaA) is one of the important virulence factors secreted by the whooping cough bacterium *Bordetella pertussis*, and it is essential for the colonization of the human respiratory tract by this bacterium. Cytotoxicity by ACT results from the synergy between toxin’s two main activities, production of supraphysiological cAMP levels by its N-terminal adenylate cyclase domain (AC domain), and cell membrane permeabilization, induced by its C-terminal pore-forming domain (hemolysin domain), which debilitate the host defenses. In a previous study we discovered that purified ACT is endowed with intrinsic phospholipase A1 (PLA) activity and that Ser in position 606 of the ACT polypeptide is a catalytic site for such hydrolytic activity, as part of G-X-S-X-G catalytic motif. Recently these findings and our conclusions have been directly questioned by other authors who claim that ACT-PLA activity does not exist. Here we provide new data on ACT phospholipase A1 characteristics. Based on our results we reaffirm our previous conclusions that ACT is endowed with PLA activity; that our purified ACT preparations are devoid of any impurity with phospholipase A activity; that ACT-S606A is a PLA-inactive mutant and thus, that Ser606 is a catalytic site for the toxin hydrolytic activity on phospholipids, and that ACT-PLA activity is involved in AC translocation.

## 1. Introduction

Phospholipases are enzymes optimized to hydrolyze phospholipids at specific ester bonds, and are classified into four major groups (PLA–D) based on the cleavage site of the ester linkage in the substrate phospholipids [1,2]. Phospholipases exist in almost every type of cell analyzed for their presence and most cells contain a multitude of them [1,2]. Phospholipases A (PLAs) cleave glycerophospholipids, the major structural lipids in eukaryotic membranes. PLA_1_s remove the fatty acid at the *sn*-1 position of the glycerol moiety, while PLA_2_s remove it at the *sn*-2 position, generating as reaction products lysophosphospholipids, which are preferentially retained in the membrane, and free fatty acids, that are more soluble and mobile with rapid flipping efficiency [1,2]. Relative to PLA_2_s which have been more extensively studied, the PLA_1_ enzymes are far less well understood, due in part to the fact that only a relatively small proportion of PLA_1_ enzymes have been cloned thus far, and no crystal structures exist for any true PLA_1_ [2]. One known common structural feature of the PLA_1_ enzymes is a lipase consensus sequence, Gly-X-Ser-X-Gly, containing the active site serine and the X denotes any amino acid [1,2].

Phospholipase A_1_ activity is conserved in a wide range of organisms and is carried out by a diverse set of PLA_1_ enzymes, though their real physiological function is not always clear enough [2]. There is numerous evidence to suggest that some PLA_1_ enzymes are virulence factors for several pathogenic bacteria [3], acting as hemolysins and cytolysins [4], very likely due to the known detergent-like properties of lysophospholids when are at high concentration [5], or acting more subtly and specifically, e.g., inhibiting autophagy and endosomal trafficking pathways [6].

The adenylate cyclase toxin (ACT or CyaA) is a key virulence factor of the whooping cough bacterium *Bordetella pertussis* [7,8,9]. ACT belongs to the Repeats-in-ToXin (RTX) family of calcium binding proteins that are secreted by many pathogenic Gram-negative bacteria through the Type I secretion system [10,11,12,13]. ACT polypeptide (1706 residues) consists of an N-terminal enzymatic adenylate cyclase (AC) domain (amino acids 1–400) that is fused to a pore-forming RTX hemolysin moiety (amino acids ≈ 501–1706) by a linker segment (amino acids 400–500) [14,15,16,17,18]. Once secreted into the medium by the producing bacteria, ACT can recognize the heterodimeric CD11b/CD18 (αMβ2) integrin), which acts as high affinity toxin receptor in myeloid phagocytic cells [19,20]. Upon binding to the integrin through its RTX domain, it is believed that ACT inserts first its hydrophobic pore-forming domain into the lipid bilayer and then delivers its N-terminal catalytic domain (AC) into the cell cytosol, crossing directly the cytoplasmic membrane and without the need for receptor-mediated endocytosis of the toxin [21,22,23]. In the host cytosol and on activation by endogenous calmodulin (CaM), it catalyzes unregulated supraphysiological-level synthesis of cAMP, which in turn alters cellular physiology and causes cytotoxic effects to the target cells [24,25].

Our understanding on the process of ACT translocation across the cell membrane is still very limited, and there are many mechanistic details of the process that remain poorly understood at the molecular level. Previous studies have shown that the translocation process is dependent on the temperature (occurring only above 15 °C), the membrane potential of the target cells, and the presence of calcium ions in the mM range [22,26,27]. Besides, it has been shown that AC domain transport is a rapid process, with a very short half-time, and that it requires structural integrity of the putative hydrophobic/amphiphatic helixes of the so-called pore-forming domain [28]. More recently, our group has made a step further in the aim of better understanding the AC translocation process. We revealed a novel calcium-dependent intrinsic phospholipase A1 activity in ACT toxin (ACT-PLA), finding that the amino acid Ser-606, located in the hydrophobic/amphipathic helical region of the toxin (pore-forming domain), is a catalytic site for such ACT-PLA activity [29,30]. Furthermore, we discovered that this lipid hydrolytic activity confers to the toxin a membrane restructuring capacity that is involved in ACT translocation, being perhaps necessary to initiate and/or favor the transport of the toxin’s AC domain across the cell membrane of target cells [29,30]. We noted that ACT-PLA is singular in several aspects, exhibiting a weak lipid hydrolytic activity relative to other potent known phospholipases A, which is not related to the ACT haemolytic-cytolytic properties [29,30], and that somehow ACT-PLA activity is not thus directly comparable to the known soluble PLA enzymes that, more or less indiscriminately, can “digest” cell membrane phospholipids and promote cell lysis, or may even be used as detergents [5].

Arguing absence of appropriate controls and simpler explanations for our reported ACT-PLA data, such as a possible contamination with *Escherichia coli* outer membrane phospholipase A (OMPLA) of the purified protein samples used in our work, our findings and conclusions of that ACT-PLA study have been directly questioned in a recent paper [31] and in a Letter [32] by the group of Dr. Sebo (Czech Academy of Sciences, Czech Republic). These investigators assure in their paper that “a highly purified and fully biologically active CyaA toxin is devoid of any detectable intrinsic PLA-1 enzyme activity.” Given the seriousness of their statements we have carefully analyzed and compared the two different experimental approaches in question [30,31]. Here we provide new data on ACT phospholipase A1 activity and its characteristics. Based on our past and present results we reaffirm here our previous conclusions that (i) ACT is endowed with intrinsic PLA activity; (ii) that our purified ACT preparations are devoid of any contaminant with phospholipase A activity; (iii) that ACT-S606A is a PLA inactive ACT mutant and that Ser-606 is a catalytic site essential for the toxin hydrolytic activity on membrane phospholipids, and (iv) that ACT-PLA activity is required for ACT translocation particularly at low, physiologically relevant toxin concentrations.

## 2. Results and Discussion

**Figure 1:**
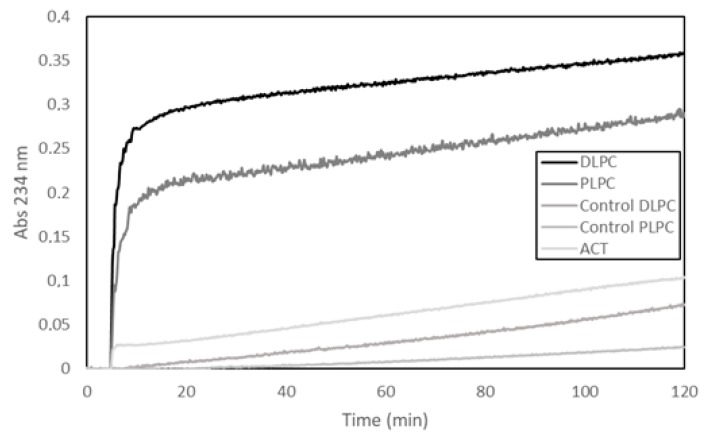
Time-course of the adenylate cyclase toxin (ACT) phospholipase A activity determined as the increase in the absorbance at 234 nm in a lipooxigenase-coupled assay. The cleavage of a fatty acid from a given phospholipid substrate (phospholipase A_1_ (PLA_1_) activity) can be indirectly determined by this coupled assay measuring the increase in absorbance at 234 nm due to the appearance of hydroperoxides by the dioxygenation of the double bonds present in the acyl chain of free fatty acids released from the phospholipid substrate. In the experiment, ACT toxin (65 nM) was added to the cuvette containing the corresponding lipid substrate (DLPC, dilauroylphosphatidylcholine, or PLPC, palmitoylphosphatidylcholine, large unilamellar vesicles (LUVs)) (65 µM) and the lipoxigenase (25 µg/mL) in the assay buffer (150 mM NaCl, 20 mM Tris, 10 mM CaCl_2_, pH = 8) 5 min after the initiation of each absorbance recording in a Varian CARY 300 Bio UV/Visible spectrophotometer. The assay temperature was 37 °C. Controls of pure lipid vesicles in buffer (DLPC or PLPC) without toxin, and of protein toxin in buffer, without lipid vesicles, are also shown.

In our previous study we used three different methods, namely, thin layer chromatography, mass spectrometry and fluorescence using a fluorogenic lipid substrate (PEDA1) specific for PLA1 activity, to determine potential phospholipase A activity in purified ACT preparations [30]. Our results from the three techniques unanimously indicated that our ACT samples had phospholipase A activity [30]. Here, we show a representative experiment, in which a fourth different methodology has been used to detect potential PLA activity, namely, a lipooxigenase-coupled spectroscopic assay. This method determines the appearance of hydroperoxides (measured as increase in Abs at 234 nm) formed by enzymatic dioxidation of double bonds in the acyl chain of free fatty acids (see Materials and Methods for details on the method), and thus, provides, indirectly, information on the putative release of free fatty acids from phospholipids by a PLA activity. In the assay shown in Figure 1, liposomes (65 µM) (large unilamellar vesicles, LUVs) composed of two different glycerophospholipids (DLPC or PLPC) were used as substrate and were incubated with ACT (65 nM) at a lipid:protein molar ratio of 1000:1 (Figure 1). The figure shows a rapid initial increase in the absorbance (Abs_234nm_) upon ACT addition to the liposomes, and consistently with the fatty acid composition of the phospholipids used in the assay (DLPC or PLPC), it is observed that the addition of ACT to the dilauroylphosphatidylcholine (DLPC) LUVs induces an absorbance increase substantially higher than that observed upon addition of the toxin to the 1-palmitoyl-2-lauroylphosphatidylcholine (PLPC) liposomes. We discarded in principle that the absorbance increase detected could be due to scattering phenomena upon toxin dilution into buffer, since exactly the same volume and same protein concentrations were added to both liposome samples (DLPC and PLPC) and the controls of different protein concentrations diluted into buffer had a negligible signal. Therefore, the data fully agree with our previous results [30] and corroborate that our purified ACT possess hydrolytic activity on phospholipids that releases free fatty acid (PLA activity).

In their Letter to Editor [32], the group of Dr. Sebo cast serious doubt on the purity of our ACT preparations, and speculated that our purified ACT samples could be contaminated with exogenous protein with phospholipase A1 activity, likely the outer membrane phospholipase A (OMPLA) coming from the *E. coli* bacteria used for ACT production [32] and, further, that this would explain the PLA1 activity detected in our assays in a simple way. To settle this issue of possible protein impurities in our toxin preparations we have performed a mass spectrometry analysis of purified ACT samples used in our ACT-PLA assays (Table S1), and as judged by the results obtained we discard, confidently, the presence of any contaminant protein with phospholipase A activity in our ACT preparations (Table S1). Moreover, and consistently with what we had already anticipated in our Response to the Letter by Masin et al. [32], we can categorically rule out the presence of the membrane protein *E. coli* OMPLA in our ACT preparations. Based on these and the above results we can reaffirm that ACT has intrinsic PLA activity.

One important observation we had insistently underlined regarding ACT was that the lipid hydrolytic activity of this toxin is faint as compared to other known more “canonical” phospholipases A [29,30]. So, while most, if not all, known and commercially available phospholipases A are soluble proteins with potent hydrolytic activities, able to more or less indiscriminately “digest” cell membrane phospholipids and promote cell lysis, or may even be used as detergents [5], ACT-PLA does not cleave all the phospholipid substrate available in the lipid bilayer. We postulated that this phenomenon could be due to a product inhibition-like effect and that might somehow constitute a kind of “life-insurance” for ACT (and for the target cell), avoiding a non-desirable degradation of the cell membrane. We hypothesized that local over-accumulation of lysophospholipids around the toxin molecules might inhibit the ACT-PLA catalytic activity, perhaps through curvature effects imposed by these lipids with intrinsic negative curvature to the lipid bilayer, hindering perhaps the insertion of the catalytic site, or other toxin segments, into the lipid bilayer and consequently halting the catalytic activity [29,30]. Hence, ACT-PLA activity shows singular features and does not seem directly comparable to other potent soluble PLA enzymes.

**Figure 2:**
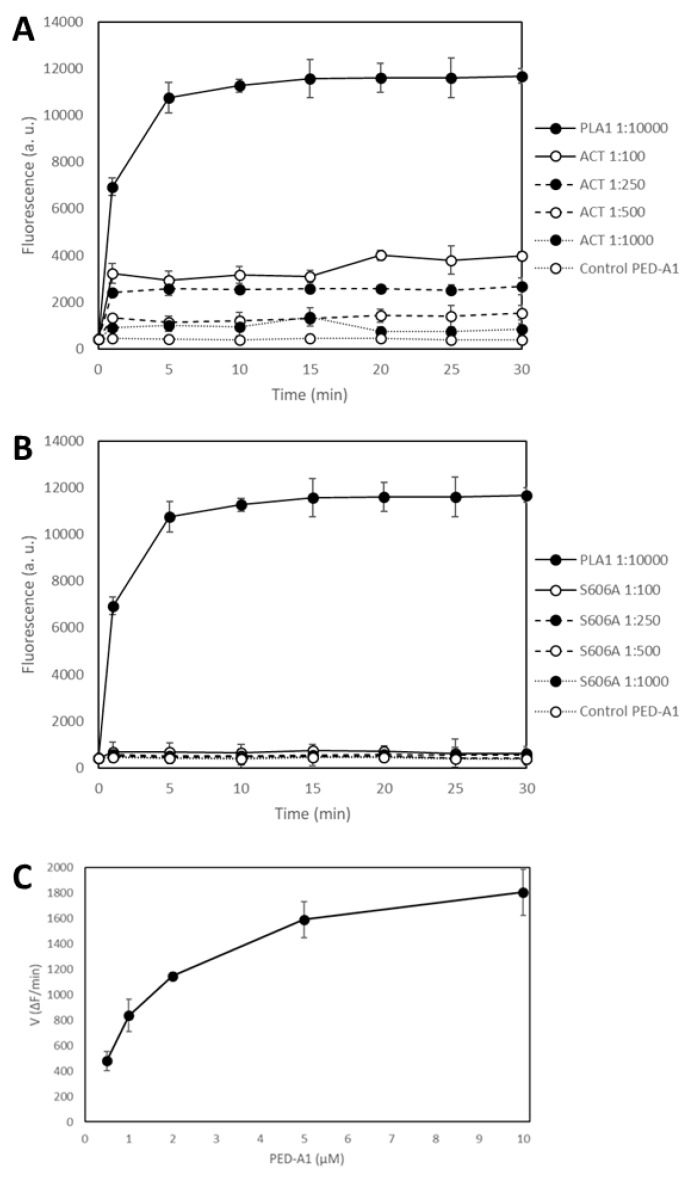
Purified adenylate cyclase toxin (ACT) cleaves the fluorogenic substrate PED-A1 (*N*-((6-(2,4-DNP)Amino)Hexanoyl)-1-(BODIPY^®^ FL C5)-2-Hexyl-Sn-Glycero-3-Phosphoethanolamine) whereas the ACT mutant ACT-S606A does not. Time-course of PLA_1_ activity determined as the increase in the fluorescence emission at 530 nm of the PLA1-specific fluorogenic substrate PED-A1 (500 nM) upon addition of wt ACT at four different concentrations (expressed as protein:lipid molar ratio), or a 1:10,000 dilution of *Thermomyces lanuginosus* phospholipase A1 enzyme stock (10 kLU/g) (**A**). Time-course of PLA1 activity determined as the increase in the fluorescence emission at 530 nm of the PLA1-specific fluorogenic substrate PED-A1 (500 nM) upon addition of the ACT mutant ACT-S606A at the same four different concentrations (expressed as protein:lipid molar ratio), or a 1:10,000 dilution of *Thermomyces lanuginosus* phospholipase A1 enzyme stock (10 kLU/g) (**B**). Fluorescence emission at 530 nm was recorded for 30 min at 37 °C, under a continuous kinetic readout in a microtiter plate, immediately upon mixing the PEDA1 fluorogenic substrate (500 nM) with the respective protein. Fluorescence data are expressed as arbitrary units (a.u) (**C**). ACT-PLA1 activity (expressed as change in fluorescence intensity per min) (∆F/min) at different fluorogenic PED-A1 lipid substrate concentrations. Data represent the mean values ± standard deviation (SD) of three independent experiments in duplicates (*n* = 6). In panels A and B, data depicted are crude fluorescence intensity values without subtracting the blank, for comparing with the intensity values of the blank itself that corresponds to the lipid substrate in buffer, without toxin. Data are depicted as the means values ± SD of three independent experiments in duplicates (*n* = 6).

With the purpose of checking whether purified ACT has or not a PLA_1_ activity and arguing lack of appropriate controls in our study, Bumba et al. [31] chose a soluble enzyme with potent PLA activity, namely, the calcium-dependent phospholipase A1 from the thermophilic fungus *Thermomyces lanuginosus* (L3295, SIGMA Aldrich) as positive control enzyme for their experiments. Determination of the lipid hydrolytic activity was performed recording fluorescence intensity of a PLA_1_-specific fluorogenic substrate, PED-A1, the same substrate we had used in our study [30]. Cleavage of the *sn*-1 ester bond of PED-A1 would induce the release of the corresponding free fatty acid, and this can be indirectly detected as an increase in the fluorescence intensity at 530 nm. Using the *Thermomyces* PLA preparations, the authors show in their paper nice time-dependent and large fluorescence intensity increases using PED-A1 (500 nM) (Figure 2A in Bumba et al [31]), which demonstrates indeed that the fluorogenic substrate is adequate for the fluorimetric detection of a PLA_1_ activity. By contrast, the changes in fluorescence intensity at 530 nm determined upon incubation of differently purified ACT preparations at a concentration of 2.5 µg/mL of toxin (≈12.5 nM) with the lipid substrate PED-A1 (500 nM) were notably smaller (inset of Figure 2B in Bumba et al [31]). For instance, the corrected intensity values (values after background subtraction) for the *Thermomyces* enzyme diluted from the stock 1:100 were ≈ 22,000 (a.u.) after 10 min of incubation, whereas the values for the different ACT samples were only of about 100–150 (a.u.), always above 0, after the same incubation time (inset of Figure 2B in Bumba and cols [31]). There is, indisputably, a big difference between both fluorescence intensity values, but, does this automatically mean that purified ACT is devoid of any detectable PLA activity? That intrinsic ACT-PLA activity does not exist? Should it not be taken into consideration that the experimental conditions used in both studies were very different? Thus, the data in Figure 2B in Bumba et al [31] would not simply indicate that, at the conditions assayed, ACT has comparatively a weak PLA activity relative to the *Thermomyces* enzyme? Given the cautions expressed clearly in our previous paper, and explained here again, regarding the faint PLA activity of ACT, and given the high specific activity of the *Thermomyces* enzyme stock, and the dilutions that were tested, were the differences in the numerical results really unexpected?

Since other issues questioned by Bumba and cols in their paper were that our previous study on ACT-PLA activity did not show crude kinetics data, and that Ser606 is a catalytic site in the ACT phospholipase A activity [31], we wanted to clarify all these doubts, and to do so we performed a PLA activity assay using, this time, the same experimental setting up that used Bumba and cols in their paper (microplate reading of fluorescence and PED-A1 at 500 nM, diluted in buffer directly from a stock solution, and not in liposomes). Figure 2 shows the time-course of PLA1 activity measured as changes in the fluorescence emission at 530 nm recorded before (*t* = 0) protein addition, and immediately after (*t* = 1 min) addition of the corresponding protein (ACT or ACT-Ser606A at different concentrations (expressed as protein:lipid molar ratio), or *Thermomyces lanuginosus* phospholipase A1 enzyme diluted 1:10,000) to the fluorogenic substrate PED-A1, and at the indicated time points for 30 min at 37 °C. The values depicted in panels A and B of Figure 2 are crude values of fluorescence intensity (a.u) recorded for each toxin concentration, and for comparison we have included the background fluorescence of the PED-A1 substrate alone in buffer. This figure shows that both the *Thermomyces* phospholipase A1 enzyme and ACT-PLA cleave the fluorogenic PED-A1 substrate, though the kinetics of lipid hydrolysis is different for both proteins, and so, while ACT-PLA cleaves the substrate very rapidly, with a high initial velocity, and then remains practically flat, the *Thermomyces* enzyme degrades the substrate progressively (Figure 2A). Figure 2 shows as well that the ACT mutant ACT-S606A does not (significantly) hydrolyze the PED-A1 substrate, and it is thus a PLA-inactive mutant. Dependency of ACT-PLA1 activity for lipid substrate concentration was also tested and as shown in Figure 2C, the lipid hydrolytic activity of ACT is saturable, with a profile similar to michaelian enzymes. From these data we estimated a K_M_ value of ≈2 µM suggesting a rather high affinity for the lipid substrate. 

Why did the group of Dr. Sebo then get such different results and conclusions? These are some explanations: (i) They used, as a positive control, large amounts of *Thermomyces* PLA, namely, 1:100 and 1:10,000 diluted preparations from the *Thermomyces* enzyme stock (specific activity >10,000 LU/g), that, according to the supplier instructions would correspond to approximately 10 and 1.0 LU/mL, respectively (One lipase unit (LU) is equivalent to the amount of lipase enzyme which releases 1 micromole of butyric acid from tributyrin per minute under the specified conditions of hydrolysis). Therefore, the authors recorded fluorescence intensity increases far larger as compared to the tiny intensity changes induced by ACT activity, this is, the values were of different magnitude, which was directly interpreted as null hydrolysis. In our Figure 2 however we show that the PLA activity of both PLA enzymes can be of the same order, and be recorded in the same or similar numerical scale, just by using more diluted *Thermomyces* PLA preparations and more concentrated ACT samples; (ii) in their experiments a (pre)-incubation of ACT with the lipid substrate occurred before fluorescence emission was recorded. Thus, they lost (or overlooked) the very rapid initial fluorescence increase that occurs upon toxin addition to PED-A1, and in consequence obtained a totally flat record (though with values >0 upon background corrections) which was again directly interpreted as null hydrolysis (inset of Figure 2B in Bumba and cols [31]). Dr. Sebo and cols have thus based their conclusions in a simple comparison of fluorescence data obtained with a single ACT concentration (no data on the PLA activity of the Ser606A mutant have been provided) and a single substrate concentration, and a soluble phospholipase A with high specific activity as positive control, and they categorically state that purified ACT is devoid of any phospholipase A enzyme activity. Our conclusions that ACT has intrinsic PLA1 activity and that ACT-S606A does not, are supported by different and abundant evidences we had provided previously and that we have reproduced and corroborated here.

A singular feature of ACT-PLA1 activity is, as we already have pointed, the rapid initial kinetics, relative to the *Thermus lanuginosus* phospholipase A. Can this be explained? Phospholipases as the fungal one are potent “soluble” enzymes with high specific activities capable of continuously degrading the lipid substrate, likely until this is consumed, and so their hydrolysis kinetics can take minutes to hours depending on the assay conditions. In contrast, ACT which associates to the membrane and inserts into the bilayer upon binding, and so, should be regarded in most aspects as an intrinsic protein in terms of membrane lateral diffusion, etc., and not as a soluble protein. This may imply from one side that the lipid hydrolysis kinetics for ACT might be conditioned by the kinetics of toxin binding and insertion into the membrane, and from other side, that the substrate availability for ACT is more reduced (only lipids surrounding toxin molecules) as compared to the any other “canonical” soluble phospholipase A. These singular ACT-PLA characteristics might explain both the fast, initial hydrolysis kinetics and the small amplitude of the fluorescence intensity changes.

**Figure 3:**
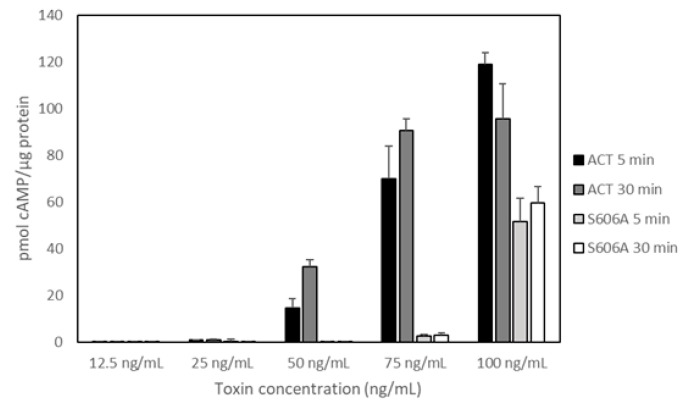
Determination of the intracellular cAMP produced by adenylate cyclase toxin (ACT) or the PLA_1_-deficient mutant ACT-S606A upon incubation with the murine macrophage J774A.1 cell line. Cells (≈1 × 10^5^ cells/mL) were incubated at 37 °C with the toxin at a final concentration in the range 0–100 ng/mL for 5 or 30 min. For determination of the intracellular cAMP the protocol provided by cAMP ELISA kit (Enzo Lifesciences) was followed. Protein concentration was determined with the Pierce BCA protein assay (Thermo Fisher Scientific). Data from three independent experiments (in duplicate, *n* = 6) are depicted and expressed as the means values ± standard deviation (SD).

Other important conclusion of our previous study [29,30,31] that Bumba and cols have questioned is whether ACT-PLA activity is or is not involved in AC translocation. To dispel any doubts, we examined the capacity of the two purified proteins to deliver the AC domain into the cytosol of target cells and elevate the cytosolic concentration of cAMP, and to do that we performed a translocation assay with ACT and the PLA-inactive mutant ACT-Ser606A using the mouse macrophage J774A.1 cell line (CD11b/CD18^+^ cells). Figure 3 shows data on the delivery of AC domain, measured as intracellular cAMP and obtained from three independent experiments in duplicates. In these experiments we used five different toxin concentrations in the range 0–100 ng/mL, identical to that used by Bumba and cols in their recent study and we tested two different incubation times, 5 and 30 min [31]. Data on toxin binding, haemolytic activity or cAMP production in solution for each protein were previously documented [30]. As shown in the figure, and relative to ACT, the capacity of the PLA-inactive mutant ACT-S606A to deliver its AC domain was practically nil for the lowest protein concentrations tested (from 12.5 to 75.0 ng/mL) at both incubation times (5 and 30 min). At the higher concentration checked (100 ng/mL), delivery of ACT-S606A achieved a value of up ≈40% to 50% of the intact ACT. These data prove that ACT-PLA activity is required for AC domain transport across the cell membrane, particularly at the low toxin concentrations that may be physiologically more relevant. Moreover, these results are fully consistent with the data published in our previous study regarding the requirement for the ACT lipid hydrolytic activity to induce cell toxicity at low ACT concentrations (Figure 8 in Reference [30]). Data shown by Bumba et al in their recent paper [31] regarding the AC delivery capacity of the PLA-inactive ACT mutant (ACT-S606A) do not agree with the results we provide here. The possible reasons for this discrepancy are not clear. These authors depicted the results as activity % giving the average value obtained for four toxin concentrations (12.5, 25, 50 and 100 ng/mL) after 30 min incubation with cells. ACT mutant ACT-D1079A, which was also questioned in the paper by Bumba et al [31] is currently under investigation in our laboratory. The results obtained will be sent for publication in a separate manuscript.

**Figure 4:**
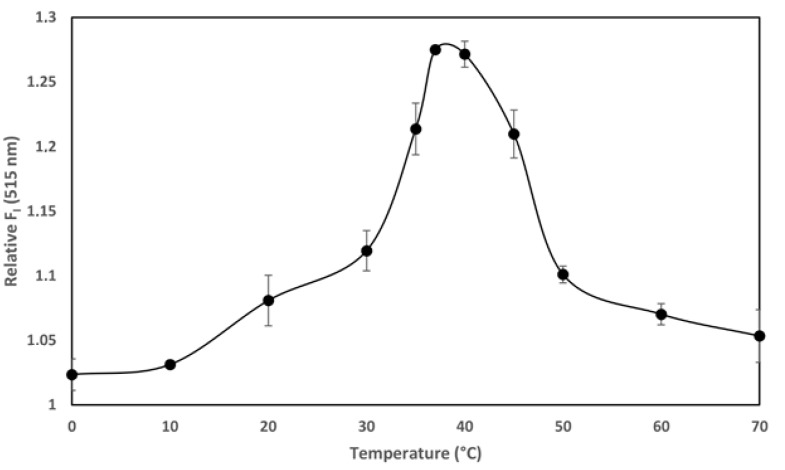
Temperature dependence of the adenylate cyclase toxin (ACT) phospholipase A_1_ activity on large unilamellar liposomes (LUVs) containing the fluorogenic phospholipid substrate PED-A1. Fluorescence emission values at 515 nm were collected for 30 min at the different incubation temperatures, upon mixing the PED-A1 fluorogenic substrate in LUVs (DOPC:PED-A1 80:20 mole ratio, 10 µM) with ACT toxin (10 nM). Fluorescence data are expressed as relative values relative to control vesicles without toxin. Data from three independent experiments (in duplicate) are depicted and expressed as the means values ± standard deviation (SD).

The temperature-dependence of the transport of the AC domain across the cell membrane (occurring only above 15 °C) is, together with its calcium-dependence at mM range, a singular hallmark of the ACT translocation process described previously in several studies [22,26,27]. We documented in our previous study that ACT is a calcium-dependent phospholipase A that requires calcium at mM concentrations for activity. Here we provide evidence of the temperature-dependence of the ACT lipid hydrolytic activity and we show that the optimum temperature for the ACT-PLA1 activity is ≈35–37 °C, and interestingly, that below ≈20 °C its lipid hydrolytic activity decreases notably (Figure 4). These data may thus nicely explain why AC translocation requires temperatures above 15 °C and strongly support our hypothesis that transport of AC across the cell membrane relies on ACT-PLA1 activity.

In conclusion, we reaffirm here our previous findings and conclusions, namely that purified ACT is endowed with intrinsic PLA activity, that Ser606 is a catalytic site for such lipid hydrolytic activity on membrane phospholipids, and importantly, that ACT PLA1 activity is involved in AC translocation, particularly at low physiologically relevant toxin concentrations.

## 3. Materials and Methods

### 3.1. Production and Purification of ACT and ACT-S606A Mutant

ACT was produced in *Escherichia coli* XL-1 blue cells (Stratagene) transformed with plasmid pT7CACT1, kindly provided by Dr. Peter Sebo (Institute of Microbiology of the ASCR, v.v.i., Prague, Czech Republic). ACT purification was performed according to the method described in Karst et al. [33]. Exponential 500 mL cultures were grown at 37 °C and induced by isopropyl-1-thio-β-d-galactopyranoside (IPTG, 1 mM) for 4h before the cells were washed with 50 mM Tris-HCl pH = 8.0, 150 mM NaCl, and disrupted by sonication. Non-broken cells were removed by centrifugation at 2500× *g* for 5 min and the supernatant was centrifuged at 25,000× *g* for 20 min. The pellet was then resuspended in 8 M urea, 50 mM Tris-HCl pH = 8.0, 50 mM NaCl, 0.2 mM CaCl_2_. Upon centrifugation at 25,000× *g* for 20 min, clarified urea extracts were submitted to several chromatrography steps as described in Karst et al. [33]. All toxins purified by this method were more than 90% pure as judged by SDS-PAGE analysis and contained less than 1 endotoxin unit of LPS/μg of protein as determined by a standard *Limulus* amebocyte lysate assay (Lonza, Biologics Porriño S.L., Barcelona, Spain). Concentrations of purified ACT proteins were determined by the Bradford assay (Bio-Rad, Hercules, CA, USA) using bovine serum albumin as standard. Protein from at least two different purifications was used in the experiments.

### 3.2. Preparation of Large Unilamellar Vesicles (LUVs)

Large unilamellar vesicles (LUVs) were prepared by extrussion of multilamellar liposomes (MLV) using polycarbonate filters of 100 nm, following the method described by Hope et al. [34].

### 3.3. In Vitro Phospholipase A Activity Assay with Fluorogenic Lipid Substrates

Highly sensitive and selective fluorogenic phospholipid substrates namely, PED-A1 (*N*-((6-(2,4-DNP)Amino)Hexanoyl)-1-(BODIPY^®^ FL C5)-2-Hexyl-Sn-Glycero-3-Phosphoethanolamine; Thermofisher Scientific, Madrid, Spain) was used to determine PLA_1_ activity. Briefly, liposomes of DOPC containing PED-A1 a 20% molar ratio (final total lipid concentration 10 µM) were incubated with the toxin (0.5–40 nM) in 20 mM Tris-HCl, 150 mM NaCl, 10 mM CaCl_2_, pH 8.0 buffer, at 37 °C under constant stirring for 30 min. Changes in fluorescence intensity at 515 nm, or fluorescence emission spectra between 490–650 nm were measured, with excitation wavelength set at 480 nm and slits at 5 nm in both monochromators.

### 3.4. In Vitro Phospholipase A Activity Assay with a Lipoxigenase-Coupled Spectroscopic Assay

DLPC (1,2-dilinoleoyl-sn-glycero-3-phosphocholine) or PLPC (1-palmitoyl-2-linoleoyl-sn-glycero-3-phosphocholine) large unilamellar vesicles were prepared as previously described in Reference [34]. Kinetic determinations were performed at 37 °C in a Varian CARY 300 Bio UV/Visible Spectrophotometer, under constant stirring. LUVs of the corresponding lipid, at a final concentration of 65 µM, and lipoxygenase (0.25 µg/mL) were incubated in buffer (150 mM NaCl, 20 mM Tris, CaCl_2_ 10 mM, pH = 8.0) and after 5′ ACT was added (65 nM). Absorbance (234 nm) was continuously recorded for 2 h.

### 3.5. Cell Culture

J774A.1 macrophages (American Type Culture Collcetion (ATCC), number TIB-67) were grown at 37 °C in Dulbecco’s Modified Eagle’s Medium (DMEM) containing 10% (*v*/*v*) fetal bovine serum FBS, and 4 mM L-glutamine in a humidified atmosphere with 5% CO_2_.

### 3.6. Measurement of cAMP

cAMP produced in cells was measured upon incubation of different ACT concentrations in the range (0–100 ng/mL) with J774A.1 cells (5 × 10^5^ cells/mL) for 5 and 30 min at 37 °C. In both cases cAMP production was calculated by the direct cAMP ELISA kit (Enzo Lifesciences, Barcelona, Spain).

## Supplementary Materials

The following are available online at http://www.mdpi.com/2072-6651/10/12/514/s1, Table S1: Mass Spectrometry Analysis of purified Adenylate Cyclase Toxin.
