# Supplementary material for: Characterization of the Intrinsic Phospholipase A1 Activity of Bordetella pertussis Adenylate Cyclase Toxin
Source: Toxins (Basel). 2018 Dec 4;10(12):514. doi: 10.3390/toxins10120514 (PMC6316389; doi:10.3390/toxins10120514)
Supplement: Supplementary file 1 [file toxins-10-00514-s001.pdf]

Accession	Description	Score	Coverage	# Proteins	# Unique Pep	# Peptides
P00350	6-phosphoglu	57.87	47.44%	1	19	19
P00509	Aspartate ami	25.08	35.61%	1	10	10
C4ZRS7	Acetyl-coenzy	6.7	10.03%	1	2	2
P24182	Biotin carboxy	5.36	5.57%	1	2	2
P0A9Q5	Acetyl-coenzy	10.87	28.95%	1	8	8
P0A9G6	Isocitrate lyas	2.39	2.53%	1	1	1
P0CK95	Putative lipop	3.36	1.25%	1	1	1
P0A6A3	Acetate kinase	5.58	6.75%	1	2	2
P25516	Aconitate hyd	12.05	6.62%	1	4	4
P36683	Aconitate hyd	38.02	21.97%	1	15	15
P21515	Acyl carrier pr	1.96	6.74%	1	1	1
P0A6A8	Acyl carrier pr	4.28	33.33%	1	2	2
P0AE06	Multidrug effl	27.19	33.75%	1	9	9
P0ACS9	HTH-type trar	14.76	26.98%	1	6	6
P27550	Acetyl-coenzy	15.92	14.57%	1	7	7
P0A9Q7	Aldehyde-alcc	27.09	15.71%	1	11	11
P0AE08	Alkyl hydrope	11.83	35.29%	1	5	5
P00561	Bifunctional a	28.69	18.29%	1	11	11
P00562	Bifunctional a	10.05	6.54%	1	4	4
P0A959	Glutamate-py	2.04	2.47%	1	1	1
P77434	Glutamate-py	7.14	13.11%	1	3	3
P25553	Lactaldehyde	7.66	10.44%	1	3	3
P0AB71	Fructose-bispl	8.03	12.81%	1	4	4
C4ZRD1	Probable cyto	6.62	12.33%	1	2	2
P04825	Aminopeptida	36.73	18.16%	1	12	12
P15034	Xaa-Pro amino	5.52	5.44%	1	2	2
P0A9E0	Arabinose ope	5.79	5.48%	1	2	2
P0A6C5	Amino-acid ac	2.8	2.03%	1	1	1
B1XBC4	Acetylglutama	1.81	3.50%	2	1	1
C5A0C3	Acetylornithir	8.67	13.32%	1	3	3
B1XHL0	Arginine repre	9.63	16.03%	1	1	1
B1XBC5	Argininosuccii	18.89	21.01%	1	8	8
P77398	Bifunctional p	72.13	23.94%	1	16	16
C4ZU95	UDP-4-amino-	20.66	24.01%	2	6	6
C4ZU96	Undecaprenyl	6.85	10.25%	1	3	3
B1X8W9	Probable 4-de	11.02	29.73%	1	5	5
P07639	3-dehydroquii	2.06	3.04%	1	1	1
P22106	Asparagine sy	2.24	1.26%	1	1	1
P0ACI6	Regulatory pr	8.14	22.37%	1	2	2
P0AC38	Aspartate ami	13.8	19.46%	1	8	8
P76216	N-succinylargi	4.66	2.46%	1	1	1
C4ZZA2	N-succinylglut	2.06	2.24%	1	1	1
P0A951	Spermidine N	3.9	5.38%	1	2	2
P0ABB0	ATP synthase	31.04	27.68%	2	9	9
B1X9W0	ATP synthase	36.08	43.26%	1	14	14
C4ZZ13	ATP synthase	2	6.21%	1	1	1
P0ABA0	ATP synthase	15.05	34.62%	1	5	5
C4ZZ11	ATP synthase	2.58	4.88%	1	1	1
P09053	Valine--pyruva	1.78	2.88%	1	1	1
P77774	Outer membr	24.22	32.91%	1	9	9

P0A903	Outer membr	28.51	38.08%	1	9	9
P0AC02	Outer membr	21.33	35.92%	1	7	7
P30843	Transcriptiona	5.46	6.76%	1	1	1
P30844	Sensor protei	6.35	6.34%	1	2	2
P37657	Cyclic di-GMP	1.88	2.29%	1	1	1
P00722	Beta-galactosi	62.01	26.37%	1	21	22
Q46829	6-phospho-be	5.07	5.01%	1	2	2
P61517	Carbonic anhy	6.91	9.55%	1	2	2
B1XBC1	Phosphoenolp	37.37	19.25%	1	15	15
P0A6F1	Carbamoyl-ph	2.13	7.59%	1	2	2
P00968	Carbamoyl-ph	49.21	26.56%	1	20	20
P21179	Catalase HPII (	2.47	2.12%	1	1	1
P0A9H7	Cyclopropane	24.43	24.87%	1	8	8
C5A1D5	60 kDa chaper	33.2	30.29%	1	12	13
C4ZU91	CinA-like prot	2.23	3.75%	1	1	1
P0ABH7	Citrate syntha	87.82	51.99%	1	16	16
P0ABH9	ATP-depender	1.83	1.58%	1	1	1
P63284	Chaperone pr	30.81	16.45%	2	11	11
B1XFM6	ATP-depender	3.48	10.61%	1	3	3
B1XHD9	tRNA U34 carl	1.97	4.02%	1	1	1
P0ABI4	Magnesium tr	8.85	14.87%	1	2	2
P0ACP1	Catabolite rep	15.74	12.57%	1	3	3
P0ACJ8	cAMP-activate	25.44	35.24%	1	7	7
P38054	Cation efflux s	3.46	2.29%	1	2	2
P16676	Sulfate/thiosu	2.35	10.96%	1	2	2
P0A9F3	HTH-type trar	18.38	20.99%	1	6	6
P0A6J1	Adenylyl-sulfa	2.84	7.46%	1	1	1
P0A9D4	Serine acetylt	16.13	20.88%	1	4	4
C4ZZR7	Sulfite reducta	10.3	8.42%	1	2	3
P0ABK5	Cysteine synth	9.08	8.67%	1	2	2
C4ZZQ5	Sulfate adeny	13.88	17.68%	1	6	6
P0AEB2	D-alanyl-D-ala	8.03	17.62%	1	5	5
B1XA76	D-amino acid	17.6	16.44%	1	5	5
P11557	Cell division p	6.31	6.78%	1	3	3
P04036	4-hydroxy-tet	3.42	7.33%	1	2	2
P0A9D8	2,3,4,5-tetrah	2.01	2.55%	1	1	1
C4ZX46	Succinyl-diam	10.99	16.27%	1	4	4
B1X701	dCTP deamina	9.87	17.10%	1	2	2
P24171	Dipeptidyl car	10.45	7.34%	1	4	4
P0AEE1	Protein DcrB (	20.69	24.32%	1	3	3
P0A9P6	ATP-depender	2.04	1.91%	1	1	1
C4ZX86	GTPase Der O	46.74	33.06%	1	12	12
P37349	Protein-lysine	4.03	4.66%	1	2	2
P0A9Q9	Aspartate-sen	11.81	13.35%	1	3	3
P00370	NADP-specific	15.58	17.90%	1	7	7
P00393	NADH dehydr	1.83	1.84%	1	1	1
P0AC41	Succinate deh	33.93	21.77%	1	9	9
P07014	Succinate deh	24.1	33.19%	1	6	6
P27296	ATP-depender	1.82	2.23%	1	1	1
P0A9P0	Dihydrolipoyl	17.32	25.74%	1	8	8
P06149	Quinone-depe	10.92	7.01%	1	4	4

P56262	Putative carbo	3.59	10.33%	1	2	2
C4ZYX9	Chromosomal	10.72	20.34%	1	8	8
P0A6Y8	Chaperone pr	77.28	42.95%	1	20	20
P00582	DNA polymera	5.52	3.02%	1	2	2
P10443	DNA polymera	18	7.50%	1	6	6
P0AAG0	Dipeptide tran	5.09	4.89%	1	1	1
C4ZTH7	1-deoxy-D-xyl	2.05	2.10%	1	1	1
P0ADB7	Entericidin B (	2.55	39.58%	1	1	1
P31545	Deferrochelat	5.86	11.58%	1	4	4
C4ZUJ5	Elongation fac	60.32	40.77%	1	19	19
P0A6P1	Elongation fac	2.11	2.83%	1	1	1
P0CE47	Elongation fac	90.34	58.88%	2	17	17
P0AEJ4	Osmolarity se	10.15	16.89%	1	5	5
P39280	L-lysine 2,3-ar	2.72	3.51%	1	1	1
P0ACL2	Exu regulon tr	5.03	12.40%	1	2	2
B1XEL4	Fructose-1,6-b	6.31	9.34%	1	2	2
B1X8Q8	3-hydroxydeca	15.17	22.09%	1	4	4
P0A953	3-oxoacyl-[acy	2.13	3.20%	1	1	1
P0AAI5	3-oxoacyl-[acy	1.96	3.39%	1	1	1
P0AEK2	3-oxoacyl-[acy	2.1	6.97%	1	1	1
P0A6R0	3-oxoacyl-[acy	2.08	4.10%	1	1	1
P0AEK4	Enoyl-[acyl-ca	5.95	12.60%	1	3	3
B1XBX4	HTH-type trar	4.31	8.37%	3	1	1
C4ZRS2	3-hydroxyacyl	3.37	10.60%	1	1	1
P10384	Long-chain fat	1.84	3.81%	1	1	1
B1XA74	Fatty acid met	4.28	14.64%	1	2	2
B1XB59	Protein FdhE (	8.48	15.53%	1	3	3
P32176	Formate dehy	46.72	19.49%	1	12	12
P0AAJ5	Formate dehy	11.98	16.67%	2	4	4
P0ABX5	Flagellar basa	1.91	4.23%	1	1	1
P0ABY7	Flagellar trans	1.86	6.25%	1	1	1
B1XGC7	Bifunctional p	7.54	11.11%	1	3	3
C4ZT96	Esterase FrsA	13.95	16.43%	1	6	6
P0A9A2	Bacterial non-	2.52	4.79%	1	1	1
P0ABH0	Cell division p	4.99	8.33%	1	2	2
C4ZZQ2	Cell division p	1.9	13.59%	1	1	1
P0AAI3	ATP-depender	19.34	13.66%	1	5	5
P10121	Signal recogni	18.73	13.48%	1	7	7
P69922	L-fucose isom	19.81	14.89%	1	5	5
P0A9S1	Lactaldehyde	1.83	2.88%	1	1	1
P0AC33	Fumarate hyd	15.14	15.15%	2	7	7
P05042	Fumarate hyd	1.98	1.93%	1	1	1
P0A9B2	Glyceraldehyc	6.64	10.88%	1	3	3
P0AC53	Glucose-6-pho	15.58	15.89%	1	8	8
B1XC24	Glucose-6-pho	19.37	17.85%	1	6	6
P25526	Succinate-sen	2.31	6.02%	1	2	2
P22256	4-aminobutyr	11	8.22%	1	2	2
P0A9S3	Galactitol 1-pl	16.05	23.12%	1	6	6
C4ZSI0	D-tagatose-1,(	15.7	26.06%	1	6	6
C4ZSH9	D-tagatose-1,(	84.8	54.05%	1	14	14
B1X7P1	GTP cyclohydr	5.42	24.32%	1	3	3

B1XEJ0	Glycine dehyd	45.06	21.11%	1	13	13
C4ZRX4	Glyoxylate/hy	8.59	11.54%	1	2	3
C4ZVX9	Glycogen synt	10.25	11.53%	1	4	4
P07762	1,4-alpha-gluc	6.01	9.07%	1	5	5
B1X775	Glucose-1-pho	12.06	19.72%	1	7	7
B1X776	Glycogen deb	1.83	1.52%	1	1	1
C4ZVS5	Glucokinase C	4.69	12.15%	1	3	3
B1XGY6	Phosphogluco	7.18	7.19%	1	2	3
P17169	Glutamine--fr	26.9	27.91%	1	13	13
P0A9C5	Glutamine syr	48.56	40.51%	1	14	14
P0A9C0	Anaerobic gly	4.43	5.54%	1	2	2
P0A996	Anaerobic gly	9.03	11.62%	1	4	4
P13035	Aerobic glycer	42.01	33.13%	1	13	13
C5A093	Glycerol kinas	48.15	27.69%	1	10	10
P52101	Sensor histidi	1.89	1.68%	1	1	1
P0AC69	Glutaredoxin	3.94	11.30%	1	1	1
C4ZXC6	Serine hydrox	9.75	19.90%	1	5	5
B1XD84	Phosphohept	9.89	18.23%	1	3	3
P32662	Phosphoglyco	2.1	2.78%	1	1	1
B1XFK5	Probable phos	4.72	8.84%	1	2	2
P25552	Guanosine-5'-	2.02	5.47%	1	2	2
P23893	Glutamate-1-s	4.69	6.57%	1	2	2
B1XCM1	Glutamate--cy	1.94	2.12%	1	1	1
P0AES0	Bifunctional g	3.92	4.85%	1	2	2
P0A9D2	Glutathione S	12.06	30.35%	1	4	4
B1XAY2	GMP synthase	19.73	17.90%	1	8	8
P0AES4	DNA gyrase su	13.12	7.89%	1	7	7
P0AES6	DNA gyrase su	20.74	16.04%	1	8	8
Q47141	Hca operon tr	2.18	4.39%	1	1	1
P15038	DNA helicase	3.04	2.19%	1	1	1
P0ACB4	Protoporphyri	4.42	11.60%	1	2	2
B1XFR2	Ferrochelatas	3.55	2.81%	1	1	1
P32131	Oxygen-indep	2.72	3.72%	1	1	1
P0ABC3	Modulator of	13.52	16.17%	1	5	5
B1XA42	High frequenc	8.52	16.90%	1	3	3
P0ABC7	Modulator of	12.07	8.59%	1	3	3
P25519	GTPase HflX C	25.63	30.52%	1	9	9
B1X6V6	ATP phosphor	6.08	20.40%	1	5	5
P06987	Histidine bios	13.51	18.03%	1	5	5
C4ZSB0	Histidinol-pho	15.62	20.79%	1	5	5
P07109	Histidine tran	2.88	4.67%	1	1	1
P0ACF8	DNA-binding	2.85	10.22%	1	1	1
P28631	DNA polymera	2.35	3.29%	1	1	1
P43329	ATP-depende	1.77	1.08%	1	1	1
P52644	Heat shock pr	5.12	15.71%	1	2	2
B1X748	33 kDa chape	22	29.11%	1	6	6
B1XB97	ATP-depende	1.86	2.03%	1	1	1
P0A6Z3	Chaperone pr	58.71	30.77%	1	17	17
B1X9B7	Small heat sh	7.22	13.87%	1	2	2
P08200	Isocitrate deh	10.97	13.46%	1	4	4
P0A705-3	Isoform Beta'	3.63	4.82%	3	3	3

C4ZYI0	Translation in	3.28	8.33%	1	1	1
P04968	L-threonine d	5.55	4.67%	1	2	2
P08142	Acetolactate s	7.45	9.43%	1	3	3
C4ZZ44	Ketol-acid red	6.15	5.50%	1	3	3
C4ZZ41	Dihydroxy-aci	4.43	4.87%	1	3	3
P0AB80	Branched-cha	2.3	3.56%	1	1	1
P0AEW6	Inosine-guanc	2.16	1.84%	1	1	1
P0CF93	Putative trans	4.89	6.76%	1	2	2
P0AAC8	Iron-binding p	2.26	13.08%	1	1	1
C4ZXA5	Cysteine desu	10.52	15.10%	1	5	5
P0AD57	Octaprenyl dip	5.33	8.36%	1	2	2
P0AEW9	1-phosphofru	1.9	5.77%	1	1	1
B1XBA8	Catalase-pero	2.33	6.75%	1	4	4
P76268	Transcriptiona	6.35	11.41%	1	3	3
P0A717	Ribose-phospl	3.85	15.87%	1	4	4
P03023	Lactose operc	9.59	15.00%	1	5	5
B1XC35	Maltoporin OS	5.84	10.09%	1	3	3
P37005	Uncharacteriz	4.65	9.21%	1	2	2
P69451	Long-chain-fa	6.76	6.77%	1	3	3
P76008	Murein tetrap	3.47	4.28%	1	1	1
P52643	D-lactate deh	6.55	8.81%	1	2	2
B1XB43	Elongation fac	41.4	32.05%	1	14	14
P00803	Signal peptida	3.7	3.09%	1	1	1
P30125	3-isopropylma	2.24	5.79%	1	2	2
C4ZXJ7	L-lactate dehy	12.55	18.94%	1	5	5
P25894	Metalloprotea	2.76	4.76%	1	1	1
P75867	Putative Lon p	15.89	13.14%	1	4	4
P45464	Penicillin-binc	14.51	10.32%	1	6	6
P0AB38	Penicillin-binc	2.11	6.57%	1	1	1
P69776	Major outer m	4.34	33.33%	1	2	2
P36771	Probable HTH	2.51	3.85%	1	1	1
P75823	Low specificity	3.26	4.20%	1	1	1
P68187	Maltose/malt	14.71	14.02%	1	4	4
B1X764	HTH-type trar	8.99	5.44%	1	5	5
P26616	NAD-depende	5.56	5.31%	1	2	2
P76558	NADP-depenc	26.66	22.79%	1	12	12
P08997	Malate syntha	2.25	3.56%	1	1	1
P0AEY3	Nucleoside tri	3.94	9.13%	1	2	2
P0ACE0	Hydrogenase-	8.03	15.70%	1	5	5
C4ZSX4	Malate dehyd	4.88	10.26%	1	3	3
P25665	5-methyltetra	4.63	3.98%	1	2	2
P13009	Methionine sy	64.18	21.52%	2	21	21
P30750	Methionine in	1.97	5.25%	1	1	1
P28635	D-methionine	13.95	28.04%	1	4	4
P30958	Transcription-	18.83	10.02%	1	9	10
P77569	DNA-binding t	2.45	6.50%	1	1	1
B1X656	tRNA-2-methy	8.01	11.60%	1	4	4
P0AEZ3	Septum site-d	1.78	5.19%	1	1	1
P0A908	MltA-interacti	3.09	5.24%	1	1	1
P76506	Probable phos	3.85	10.36%	1	3	3
P50456	Protein mlc O	4.11	7.88%	1	3	3

C4ZZ19	tRNA uridine !	4.98	3.34%	1	1	1
P12281	Molybdopteri	3.1	4.38%	1	1	1
P37773	UDP-N-acetyl	2.58	2.41%	1	1	1
P0ACR9	Transcriptiona	4.19	9.09%	1	2	2
B1X6C4	Protein MtfA (	5.03	10.94%	1	2	2
P40874	N-methyl-L-tr	2.39	3.49%	1	1	1
C4ZQ50	Chromosome	25.64	9.76%	1	13	13
P22524	Chromosome	8.71	17.95%	1	2	2
C4ZQ48	Chromosome	17.64	23.64%	1	7	7
C4ZSS8	UDP-N-acetylg	3.9	5.01%	2	2	2
P22188	UDP-N-acetyl	3.63	5.05%	1	1	1
B1XC67	UDP-N-acetylg	1.82	3.38%	1	1	1
B1XCR0	DNA mismatc	9.29	6.33%	1	5	5
P27278	Trifunctional I	1.98	2.93%	1	1	1
C4ZU31	Nucleoid-asso	24.2	22.69%	1	5	5
P0ADA3	Murein hydro	2.34	6.86%	1	2	2
P40710	Lipoprotein N	2.32	19.07%	1	3	3
C4ZSQ4	Lipoprotein N	6.56	12.24%	1	3	3
B1XCN6	Anaerobic nit	2.45	2.18%	1	1	1
P0AFB8	DNA-binding t	1.84	2.13%	1	1	1
B1XA44	Phosphatase I	3.02	11.11%	1	1	1
C4ZUD0	NADH-quinon	14.75	16.82%	1	3	3
P33599	NADH-quinon	62.46	41.95%	2	19	19
P31979	NADH-quinon	24.36	21.80%	1	9	9
P33602	NADH-quinon	30.27	15.97%	1	10	10
P0AFD6	NADH-quinon	16.17	35.56%	1	5	5
P0AFF6	Transcription	60.43	37.98%	1	16	16
B1XHF9	GTPase Obg C	2.5	5.90%	1	1	1
P0AFG3	2-oxoglutarat	58.89	32.15%	1	21	21
P0AFG6	Dihydrolipoyll	23.53	25.19%	1	9	9
P0AFG8	Pyruvate deh	71.2	32.13%	1	22	22
P06959	Dihydrolipoyll	148.01	49.21%	1	26	26
P0A910	Outer membr	15.24	21.68%	1	5	5
P0AA16	Transcriptiona	10.6	22.18%	1	4	4
P27298	Oligopeptidas	41.45	22.21%	1	11	11
P76027	Oligopeptide t	5.48	14.84%	1	3	3
P77737	Oligopeptide t	15.17	24.85%	1	6	6
P0ADB1	Osmotically-ir	6.94	38.39%	1	3	3
P0A9L8	Pyrroline-5-ca	2.02	3.35%	1	1	1
P0A912	Peptidoglycan	19.99	32.37%	1	4	4
B1XCA8	Pantothenate	2.26	14.13%	1	2	2
P0AFI2	DNA topoison	7.53	8.11%	1	4	4
P20083	DNA topoison	16.88	14.60%	1	7	7
B1X750	Phosphoenolp	1.9	5.74%	1	2	2
B1XAZ8	Peptidase B O	3.86	5.15%	1	2	2
P15288	Cytosol non-s	26.65	21.03%	1	8	8
B1XAK9	Xaa-Pro dipep	2.28	3.16%	1	1	1
P09373	Formate acety	69.19	29.47%	2	16	16
P36938	Phosphogluco	3.88	6.04%	1	1	3
P0A9J8	P-protein OS=	3.43	3.89%	1	2	2
P0A9K3	PhoH-like pro	4.31	6.36%	1	1	2

P0A9K7	Phosphate-sp	36.18	48.13%	1	9	9
P0AC86	Glycogen pho	4.91	3.56%	1	2	2
P00490	Maltodextrin	42.2	22.58%	1	14	14
C4ZSQ5	Polyribonucle	6.34	9.70%	1	5	5
P69874	Spermidine/p	4.61	6.88%	1	2	2
P23869	Peptidyl-proly	4.26	19.51%	1	2	2
P0ADY1	Peptidyl-proly	74.58	41.25%	1	16	16
P0A7B1	Polyphosphat	32.55	18.60%	1	11	11
P23538	Phosphoenolp	42.64	29.29%	1	17	17
P0AFL6	Exopolyphosp	8.87	10.33%	1	4	4
P0A8T1	Ribosomal prc	1.9	5.12%	1	1	1
P07004	Gamma-gluta	2.66	3.12%	1	1	1
C4ZT99	Glutamate 5-k	9.04	10.63%	1	3	3
C4ZZI8	RNA chaperor	2.81	4.31%	1	1	1
P23830	CDP-diacylgly	3.7	5.54%	1	1	1
P37177	Phosphoenolp	12.9	11.63%	1	6	6
P08839	Phosphoenolp	28.5	22.43%	1	11	11
P0A9M8	Phosphate ac	11.05	8.82%	1	5	5
P69828	PTS system ga	29.88	48.67%	1	4	4
P37188	PTS system ga	4.21	20.21%	1	1	1
P0AG16	Amidophosph	24.8	18.81%	1	7	7
P08179	Phosphoribos	6.86	17.45%	1	3	3
P15254	Phosphoribos	36.72	16.76%	1	15	15
B1XAX4	Phosphoribos	2.16	4.35%	1	1	1
B1XAE8	Phosphoribos	10.79	16.88%	1	4	4
P0AB89	Adenylosuccir	9.74	9.43%	1	3	3
B1XDS8	Adenylosuccir	15.2	22.69%	1	7	7
C4ZYC2	HTH-type trar	7.34	13.20%	1	3	3
P33221	Formate-depe	11.4	17.60%	1	4	4
P37051	Formyltetrahy	4.31	5.36%	1	1	1
P09546	Bifunctional p	34.34	16.89%	1	15	15
P78061	Gamma-gluta	10.45	12.71%	1	5	5
P0A7E5	CTP synthase	9.89	8.07%	1	4	4
P0AA53	Protein QmcA	7.75	17.38%	1	3	4
P24554	DNA repair pr	4.61	5.87%	1	3	3
C4ZPY3	RNA polymera	38.98	21.59%	1	16	16
P31473	ATPase RavA (	7.01	6.02%	1	3	3
P21893	Single-strande	18.13	17.16%	1	8	8
P0AG20	GTP pyrophos	6.3	5.38%	1	3	3
B1XEH8	Peptide chain	18.48	19.73%	1	7	7
P0A7I4	Peptide chain	8.04	9.07%	1	2	3
C4ZZ48	ATP-depende	4.49	11.16%	1	3	3
P0AG30	Transcription	9.88	14.08%	1	6	6
P0AG40	Bifunctional ri	3.25	7.67%	1	1	1
B1X7X6	Ribosomal prc	8.18	10.88%	1	4	4
P00452-2	Isoform Alpha	30.53	14.81%	2	9	9
P69924	Ribonucleosid	19.35	25.80%	1	5	5
B1XBY7	50S ribosoma	6.11	24.85%	1	3	3
C5A0S3	50S ribosoma	2.18	7.04%	1	1	1
P0AA10	50S ribosoma	2	4.93%	1	1	1
C4ZUG5	50S ribosoma	1.8	16.26%	1	1	1

P02413	50S ribosoma	19.34	33.33%	1	4	4
C4ZUG8	50S ribosoma	5.73	22.06%	1	2	2
B1X6E6	50S ribosoma	7.92	20.47%	1	3	3
C4ZUF9	50S ribosoma	1.99	7.69%	1	1	1
P0A7K6	50S ribosoma	1.89	6.96%	1	1	1
B1XG23	50S ribosoma	4.09	17.80%	1	2	2
B1X6G8	50S ribosoma	10.55	17.22%	1	4	4
B1X6F4	50S ribosoma	1.86	13.56%	1	1	1
C4ZYH9	50S ribosoma	2.56	20.00%	1	1	1
P60723	50S ribosoma	1.8	7.46%	1	1	1
B1X6G0	50S ribosoma	49.64	64.80%	1	12	12
B1X6F7	50S ribosoma	4.11	25.99%	1	3	3
P0C0R7	Ribosomal RN	2.22	4.31%	1	1	1
C4ZQ93	Ribosomal RN	3.38	4.04%	1	1	1
P36979	Dual-specificit	7.27	6.51%	1	2	2
P33643	Ribosomal lar	15.78	23.93%	1	4	4
C4ZTY2	Exoribonuclea	8.24	7.61%	1	4	4
P0A7Y0	Ribonuclease	5.5	7.52%	1	2	2
P21513	Ribonuclease	157.79	38.17%	1	30	30
P0CG18	Ribonuclease	5.56	11.34%	2	2	2
P21499	Ribonuclease	3.85	3.44%	1	2	2
P27434	Cytoskeleton	1.89	3.86%	1	1	1
P24255	RNA polymera	5.59	6.71%	1	3	3
P0ACS7	HTH-type trar	6.87	14.19%	1	3	3
P0A7Z4	DNA-directed	15.59	21.58%	1	6	6
C5A0S7	DNA-directed	253.36	57.23%	1	60	60
B1XBZ0	DNA-directed	71.56	27.08%	1	26	27
P00579	RNA polymera	36.52	18.11%	1	11	11
P13445	RNA polymera	9.75	14.85%	1	4	4
B1XB95	Regulator of r	8.26	14.29%	1	3	3
C4ZUF2	30S ribosoma	1.9	9.30%	1	1	1
C4ZUJ7	30S ribosoma	6.82	8.87%	2	2	2
P0A7S9	30S ribosoma	10.13	33.05%	1	4	4
P0AG59	30S ribosoma	2.81	9.90%	1	1	1
C4ZUG6	30S ribosoma	1.79	9.52%	1	1	1
C4ZR79	30S ribosoma	7.97	40.00%	1	3	3
P0AG67	30S ribosoma	8.71	9.87%	1	4	4
C4ZRR1	30S ribosoma	21.81	37.34%	1	6	6
P0A7V3	30S ribosoma	5.3	14.59%	1	3	3
B1X6E8	30S ribosoma	15.25	26.21%	1	5	5
P0A7W1	30S ribosoma	1.93	11.38%	1	1	1
C4ZR77	30S ribosoma	5.34	26.72%	2	4	4
B1X6F8	30S ribosoma	1.99	6.15%	1	1	1
B1XHK3	30S ribosoma	3.46	26.15%	1	3	3
B1X6E1	Ribosomal RN	9.68	14.22%	1	4	4
P38104	Starvation-ser	1.91	2.97%	1	1	1
P52108	Transcriptiona	11.3	14.64%	1	4	4
P30744	L-serine dehy	2.14	1.98%	1	1	1
P10408	Protein transl	17.95	7.99%	1	6	6
P0AG86	Protein-expor	10.78	28.39%	1	3	3
P0A9T0	D-3-phosphog	36.54	27.56%	1	10	10

P0AGB0	Phosphoserin	6.98	18.63%	1	4	4
B1X847	Phosphoserin	2.38	8.84%	1	3	3
P0AGM5	UPF0162 prot	12.78	29.00%	1	5	5
P0C093	Nucleoid occlu	5.49	13.13%	1	2	2
P0A905	Outer membr	2.26	7.74%	1	1	1
P0AGD3	Superoxide di	3.39	7.25%	1	1	1
P21170	Biosynthetic a	5.28	4.26%	1	2	2
P0AG24	Bifunctional (|	4.05	3.56%	1	3	3
P0AGD7	Signal recogni	13.62	18.10%	1	5	5
P0AFZ3	Stringent starv	2.21	6.67%	1	1	1
P76072	Prophage side	2.64	1.43%	1	1	1
C5A0R1	Soluble pyridi	7.39	8.15%	1	3	3
B1X6Q8	Succinate--Co	48.44	38.14%	1	11	11
P0AGE9	Succinate--Co	15.43	26.64%	1	6	6
P77522	FeS cluster as	1.81	3.23%	1	1	1
P0ADG4	Inositol-1-mor	3.54	8.24%	1	2	2
P00957	Alanine--tRNA	67.75	39.61%	1	25	25
B1XGC4	Cysteine--tRN	7.86	11.93%	1	3	3
B1XHD4	Aspartate--tRI	16.77	16.10%	1	9	9
B1X9R9	Glutamate--tF	2.41	6.79%	1	2	2
P08312	Phenylalanine	5.78	10.70%	1	3	3
P07395	Phenylalanine	26.81	17.23%	1	8	8
C4ZXF0	Glycine--tRNA	5.5	9.90%	2	2	2
B1X8H5	Glycine--tRNA	86.63	40.20%	1	23	23
P60906	Histidine--tRN	20.15	22.88%	1	7	7
C4ZPV2	Isoleucine--tR	26.66	14.71%	1	12	12
P0A8N3	Lysine--tRNA l	37.05	34.85%	1	10	13
P0A8N5	Lysine--tRNA l	41.3	33.86%	1	10	13
P07813	Leucine--tRNA	30.18	20.47%	1	14	14
C4ZSJ8	Methionine--t	33.77	20.24%	1	9	9
P0A8M0	Asparagine--tI	32.08	24.25%	1	9	9
C4ZWF6	Glutamine--tR	1.81	3.07%	1	2	2
B1XHE4	Arginine--tRN	4.33	6.59%	1	3	3
B1X833	Serine--tRNA l	11.43	18.60%	1	6	6
B1XGI1	Threonine--tR	30.5	19.31%	1	11	11
P07118	Valine--tRNA l	59.05	23.24%	1	19	19
P00954	Tryptophan--t	2.42	5.09%	1	1	1
P0AGJ9	Tyrosine--tRN	3.58	4.48%	1	2	2
P08957	Type I restrict	7.02	4.91%	1	2	3
P08956	Type I restrict	12.71	8.12%	1	7	7
P0A870	Transaldolase	44.88	58.04%	2	14	14
P69428	Sec-independ	3.29	14.61%	1	1	1
P69425	Sec-independ	2.55	14.04%	1	2	2
C4ZXK8	L-threonine 3-	5.86	7.04%	1	2	2
C4ZTI0	tRNA sulfurtra	4.07	8.09%	1	2	3
P31142	3-mercaptopy	3.64	4.27%	1	1	1
B1XFM4	Trigger factor	7.94	12.73%	1	4	4
P27302	Transketolase	23.7	12.37%	1	6	6
P19934	Tol-Pal system	2.06	6.89%	1	2	2
P18004	Protein TraC (	2.06	0.80%	1	1	1
P0AE01	tRNA (cytidine	2.35	6.50%	1	1	1

P0A879	Tryptophan sy	1.94	3.02%	1	1	1
P07649	tRNA pseudou	5.71	14.07%	1	3	3
C4ZZP9	tRNA pseudou	4.19	6.02%	1	2	2
P32132	GTP-binding p	2.08	1.81%	1	1	1
P07023	T-protein OS=	5.22	6.17%	1	2	2
P07604	Transcriptiona	4.67	4.68%	1	2	2
P0A884	Thymidylate s	7.58	14.77%	1	3	3
P75728	2-octaprenyl-3	2.75	3.32%	1	1	1
P76373	UDP-glucose 6	3.58	8.25%	1	3	3
P0A8F0	Uracil phosph	2.39	8.17%	1	1	1
P0AED0	Universal stre	8.84	40.97%	1	2	2
P43672	ABC transport	16.65	13.07%	1	7	7
P0A698	UvrABC syster	7.61	8.19%	1	5	5
C4ZXV1	UvrABC syster	31.27	22.14%	1	11	11
P03018	DNA helicase	1.81	1.67%	1	1	1
P39161	Uxu operon tr	2.2	3.89%	1	1	1
B1X9X3	Protein ViaA (	3.72	3.93%	1	2	2
C4ZZ62	UDP-N-acetyl-	3.76	5.28%	1	1	1
P76372	Chain length c	4.46	6.44%	1	2	2
B1XAH7	Tyrosine recoı	8.42	14.09%	1	4	4
P0A8P8	Tyrosine recoı	8.39	12.42%	1	3	3
Q47147	Putative gluta	2.17	3.92%	1	1	1
P0AA99	Putative L,D-t	1.91	7.72%	1	1	1
P77169	Putative unch	1.79	4.53%	1	1	1
P0ADA5	Uncharacteriz	2.29	7.29%	1	1	1
P0ACJ5	DNA-binding t	3.56	6.58%	1	1	1
P0AFP4	Uncharacteriz	2.33	8.18%	1	1	1
B1X654	Endoribonucle	4.56	9.68%	1	1	1
P46130	Putative acyl-(	5.14	6.09%	1	2	2
C4ZXW7	UPF0194 men	13.03	21.99%	1	4	4
P30178	Hydroxycarbo	9.55	18.28%	1	4	4
P0ACU0	HTH-type trar	7.6	15.70%	1	3	3
P0A9U3	Uncharacteriz	9.54	8.87%	1	4	4
P75838	Ribosomal prc	11.85	14.85%	1	5	5
P75915	Chaperone pr	12.5	34.78%	1	5	5
P24188	UPF0176 prot	2.51	4.00%	1	1	1
P27431	50S ribosoma	9.27	10.46%	1	3	3
P0ABU2	Ribosome-bin	3.22	6.06%	1	2	2
P0A8Z0	Acyl-CoA thio	6.54	29.55%	1	3	3
P64451	Uncharacteriz	2.35	11.26%	1	1	1
P76104	Uncharacteriz	9.19	5.82%	1	4	4
P77156	Inner membra	1.81	5.11%	1	1	1
P67699	Uncharacteriz	1.81	15.96%	1	1	1
P77804	Protein YdgA (	24.96	28.69%	1	10	10
P77748	Uncharacteriz	36.32	16.80%	1	14	14
P76245	Diguanylate cy	11.2	20.23%	1	4	4
P0A8A0	Probable tran	2.16	4.07%	1	1	1
P76403	Uncharacteriz	20.59	21.41%	1	6	6
P33355	Uncharacteriz	2.73	8.97%	1	1	1
P33029	Uncharacteriz	4.11	6.15%	3	3	3
P76507	Uncharacteriz	1.81	1.81%	1	1	1

P76578	Uncharacteriz	2.46	0.60%	1	1	1
P33634	Uncharacteriz	2.03	3.75%	1	1	1
P0ADR8	Pyrimidine/pu	37.14	32.38%	1	11	11
P65294	Uncharacteriz	2.13	12.50%	1	1	1
P64574	Uncharacteriz	2.07	12.63%	1	1	1
P0ADT5	Putative acid-	2.27	2.33%	1	1	1
P30871	Inorganic tripl	6.22	4.39%	1	2	2
P0A894	RNase adapte	6.65	11.27%	1	2	2
P0ADV5	Luciferase-like	2.24	8.06%	1	2	2
P0ADW3	Inner membra	9	15.91%	1	2	2
P64624	Uncharacteriz	10.05	17.92%	1	2	3
P63389	Uncharacteriz	6.01	5.02%	1	2	2
P46837	Protein YhgF (	17.98	13.58%	1	7	7
P37627	Uncharacteriz	1.96	1.85%	1	1	1
P0ADK0	Uncharacteriz	16.45	20.76%	1	4	4
P11290	Uncharacteriz	4.96	6.40%	1	2	2
C4ZYY3	Membrane pr	2.94	5.11%	1	1	1
P31475	Uncharacteriz	3.78	9.13%	1	1	1
P23305	Uncharacteriz	7.86	25.53%	1	4	4
B1XAM2	Der GTPase-ac	6.98	17.75%	1	3	3
P0A8Y3	Alpha-D-gluco	5.77	7.04%	1	1	1
P0ADQ2	Uncharacteriz	2.89	4.56%	1	1	1
P32680	Uncharacteriz	7.68	19.39%	1	3	3
P0ACU7	HTH-type trar	6.84	26.18%	1	4	4
P0AF70	Uncharacteriz	6.48	30.77%	1	2	2
P24203	P-loop guanos	2.5	3.14%	1	1	1
P0A8Y1	Pyrimidine 5'-	2.74	6.22%	1	1	1
P0A9W3	Energy-depen	11.22	11.35%	1	4	4
P64429	Uncharacteriz	3.73	8.36%	1	2	2
Q46939	Probable acet	5.96	10.94%	1	3	3
Q46855	Uncharacteriz	1.86	3.46%	1	1	1
P64596	Uncharacteriz	13.09	33.51%	1	5	5
P0A9W9	Protein YrdA (	4.27	11.96%	1	2	2
Q9JMS5	Uncharacteriz	1.87	0.74%	1	1	1
P36680	Cell division p	7.56	14.98%	1	3	3
C4ZV90	Zinc transport	3.63	11.93%	1	3	3
P0ACS5	HTH-type trar	3.87	13.48%	1	2	2
P00761	Trypsin OS=Su	34.51	31.17%	1	5	5
P0DKX7	Bifunctional h	11218.47	85.17%	1	138	138
P11553	L-fuculokinase	28.8	28.18%	1	10	10
P06720	Alpha-galacto	0	2.44%	1	1	1
P0AE12	AMP nucleosi	0	2.27%	1	1	1
P69503	Adenine phos	2.18	7.65%	1	1	1
P24240	6-phospho-be	1.73	6.54%	1	2	2
P0AE37	Arginine N-su	0	5.52%	1	1	1
B1XGK5	Succinylglutar	0	3.42%	1	1	1
P68699	ATP synthase	1.61	20.25%	1	2	2
P30847	Signal transdu	1.61	3.64%	1	1	1
P37652	Cyclic di-GMP	0	3.85%	1	1	2
P17445	NAD/NADP-de	3.26	3.27%	1	1	2
P05804	Beta-glucuron	1.65	1.33%	1	1	1

B1X7A5	Biotin synthas	1.63	5.49%	1	1	1
P33927	Cytochrome c	1.62	2.47%	1	1	1
P39163	Glutathione-s	0	3.90%	1	1	1
P0A6G7	ATP-depende	2.16	8.70%	1	1	1
P0AE78	Magnesium a	1.67	4.45%	1	1	1
P0AE82	Sensor histidi	3.67	6.35%	1	1	2
B1XDY3	Sigma factor-I	1.63	6.77%	1	1	1
P00936	Adenylate cyc	3.38	2.83%	1	2	2
P0AEB0	Sulfate transp	3.32	2.41%	1	1	1
P29680	Uroporphyrin	5.47	12.71%	1	3	3
B1XFJ3	Phosphopent	0	4.91%	1	1	1
P31460	Galactonate o	2.22	10.04%	1	1	1
P76015	PEP-depende	0	4.49%	1	1	1
P69853	Tat proofread	1.67	6.37%	1	1	1
C4ZPU1	Chaperone pr	0	2.66%	2	1	1
P06710	DNA polymer	6.77	7.62%	2	4	4
B1X7E2	DNA protectic	0	5.39%	1	1	1
P0AB83	Endonuclease	1.73	5.21%	1	1	1
P37690	Murein hydro	0	5.01%	1	1	1
B1XD26	Iron-sulfur clu	1.68	10.53%	1	1	1
P0ACZ4	DNA-binding t	0	6.86%	1	1	1
P21151	3-ketoacyl-Co	0	4.13%	1	1	1
B1XFC2	Probable Fe(2	1.75	17.58%	1	1	1
P0AEN1	NAD(P)H-flavi	1.65	12.02%	1	2	2
P46889	DNA transloc	0	1.13%	1	1	1
P09148	Galactose-1-p	0	3.16%	1	1	1
P0A9I3	Glycine cleav	2.28	6.84%	1	1	1
Q46839	Glycolate per	0	2.86%	1	1	1
P27249	Bifunctional u	2.08	1.57%	1	1	1
P09394	Glycerophosp	1.64	3.07%	1	1	1
P75796	Glutathione ir	0	1.61%	1	1	1
P09126	Uroporphyrin	0	2.85%	1	1	1
P09127	Protein HemX	1.66	5.34%	1	2	2
P23873	Antitoxin HipI	0	13.64%	1	1	1
P0CF55	Transposase I	0	3.32%	2	1	1
P39347	Putative prote	0	3.28%	1	1	1
P24218	Prophage inte	0	3.62%	1	1	1
P75969	Prophage inte	2.25	4.27%	1	1	1
P76056	Prophage inte	1.61	2.43%	1	1	1
B1XB82	ATP-depende	1.63	2.81%	1	1	1
P0AB77	2-amino-3-ke	1.66	6.78%	1	2	2
C4ZTQ6	2-dehydro-3-c	1.61	3.52%	1	1	1
P0AEX5	Probable pho	1.68	2.42%	1	1	1
C4ZPZ5	3-isopropylm	2.19	4.08%	1	1	1
P30015	Probable ATP-	1.61	0.59%	1	1	1
C4ZTQ0	Outer-membr	3.28	11.59%	1	1	2
P0A9V1	Lipopolysacch	1.62	4.98%	1	1	1
C4ZWC8	LPS-assembly	0	13.99%	1	2	2
P21645	UDP-3-O-(3-h	2.22	4.11%	1	1	1
P15977	4-alpha-gluca	5.18	6.20%	1	4	4
P24174	Mannose-1-pl	0	2.51%	1	1	1

P37330	Malate syntha	0	1.24%	1	1	1
P15005	5-methylcytos	0	1.96%	1	1	1
P37637	Multidrug resi	1.64	1.64%	1	1	1
P0A935	Membrane-bo	2.19	2.74%	1	1	1
P0A9X4	Rod shape-de	0	4.32%	1	1	1
P60752	Lipid A export	2.06	3.09%	1	1	1
P10902	L-aspartate ox	0	2.78%	1	1	1
B1X8A2	Periplasmic ni	0	1.69%	1	1	1
P38489	Oxygen-insens	1.71	5.07%	1	1	1
P08201	Nitrite reducta	1.77	3.78%	1	1	2
P31061	Prophage DNA	2.14	11.64%	1	1	1
P37596	Nitric oxide re	0	4.51%	1	1	1
P0AFD1	NADH-quinon	1.67	4.82%	1	1	1
C4ZRY9	Glucans biosy	0	2.54%	1	1	1
P0C0L2	Peroxiredoxin	1.71	7.69%	1	1	1
P31677	Trehalose-6-p	1.63	2.11%	1	1	1
P28305	Aminodeoxyc	0	6.32%	1	1	1
P0AFI5	D-alanyl-D-ala	3.81	13.55%	1	2	2
C4ZVL3	Erythronate-4	0	3.17%	1	1	1
P00914	Deoxyribodipy	1.75	2.54%	1	1	1
P0C0L7	Proline/betair	1.74	2.20%	1	1	1
C5A1F4	Phosphatidyls	2.13	3.42%	1	1	1
P37344	Psp operon tra	0	3.69%	1	1	1
C4ZYG5	Phosphoenolp	0	9.39%	1	2	2
P32676	PTS system fru	0	14.16%	1	1	1
P77439	Multiphospho	1.7	2.05%	1	1	1
P23883	NADP/NAD-de	2.04	2.42%	1	1	1
B1XEZ3	S-adenosylme	2.13	5.62%	1	1	1
P03856	Replication in	1.61	3.59%	1	1	1
P0A7I0	Peptide chain	1.64	1.94%	1	1	1
P25741	Lipopolysacch	0	5.66%	1	1	1
C4ZWD8	Pyrimidine-sp	3.36	7.40%	1	2	2
P0A7L0	50S ribosoma	0	5.56%	1	1	1
B1XBA2	50S ribosoma	0	10.00%	1	1	1
B1X6H1	50S ribosoma	0	3.83%	1	1	1
P10100	Endolytic pep	2.11	3.04%	1	1	1
P0A9J0	Ribonuclease	1.67	3.07%	1	1	1
P0ACI0	Right origin-bi	0	3.46%	1	1	1
P0AGB3	RNA polymera	1.67	2.46%	1	1	1
C4ZSQ6	30S ribosoma	1.74	7.87%	1	1	1
B1XG70	30S ribosoma	0	11.27%	1	1	1
P16456	Selenide, wate	1.73	3.75%	1	1	1
B1XFT8	tRNA 2-selenc	0	1.92%	1	1	1
P39365	Putative perm	0	2.06%	1	1	1
P39364	Putative sgc re	0	4.85%	1	1	1
P0A9E2	Regulatory pro	2.45	15.89%	1	1	2
C4ZRL3	S-adenosylme	1.61	5.68%	1	1	1
P0AGE0	Single-strande	1.74	5.62%	1	1	1
C4ZYN7	SsrA-binding p	1.7	5.00%	1	1	1
P0ABZ6	Chaperone Su	0	3.27%	1	1	1
B1XD64	Proline--tRNA	2.16	4.55%	1	2	2

C5A0T1	Thiazole synth	1.73	4.30%	1	1	1
P0AGG0	Thiamine-mor	1.66	4.31%	1	1	1
P00934	Threonine syr	2.1	2.34%	1	1	1
Q00042	Putative trans	2.11	3.15%	1	1	1
P06612	DNA topoison	3.38	2.08%	1	2	2
P38683	Periplasmic pr	2.02	4.68%	1	1	1
P41067	Protein TraB (	0	4.42%	1	1	1
P14497	Protein TraF (	1.68	4.45%	1	1	1
B1XAN8	Periplasmic tr	0	1.95%	1	1	1
P60340	tRNA pseudou	0	3.50%	1	1	1
P0AA41	tRNA pseudou	0	5.00%	1	1	1
P0AAB4	3-octaprenyl-4	0	1.61%	1	1	1
P04152	Protein UmuC	1.63	3.08%	1	1	1
P0A8F4	Uridine kinase	0	4.69%	1	1	1
P0AAC0	Universal stre	1.73	2.85%	1	1	1
P37751	Putative glyco	1.69	2.96%	1	1	1
P27828	UDP-N-acetylg	1.69	1.86%	1	1	1
P39220	Putative unch	2.03	7.87%	1	1	1
P37056	Probable lipop	0	5.47%	1	1	1
P0AA97	Uncharacteriz	0	3.87%	1	1	1
P30864	Uncharacteriz	1.7	3.29%	1	1	1
B1XD73	UPF0294 prot	0	3.38%	1	1	1
P77393	Uncharacteriz	1.66	7.75%	1	2	2
P77216	Uncharacteriz	1.72	2.46%	1	1	1
P0AAV4	Uncharacteriz	1.73	5.50%	1	1	1
P75764	Uncharacteriz	2.02	1.46%	1	1	1
P75783	Moderate cor	0	1.62%	1	1	1
C4ZY19	Putative trans	2.15	3.21%	1	1	1
P0AAY6	Uncharacteriz	1.62	7.59%	1	1	1
P75818	Uncharacteriz	1.63	6.43%	1	1	1
P75821	Uncharacteriz	0	4.45%	1	1	1
P75829	Uncharacteriz	1.73	3.33%	1	1	1
P22525	Probable L,D-t	0	3.09%	1	1	1
P77552	Uncharacteriz	1.69	1.91%	1	1	1
P76393	Uncharacteriz	1.68	1.85%	1	1	1
P76418	Uncharacteriz	0	5.69%	1	1	1
P33341	Outer membr	0	2.30%	1	2	2
P33342	Probable fimb	0	4.18%	1	1	1
C4ZVI4	5'-deoxynucle	0	4.52%	1	1	1
P76573	Uncharacteriz	0	5.03%	1	1	1
P0AD44	Uncharacteriz	1.7	5.49%	1	1	1
P52124	Uncharacteriz	0	3.62%	1	1	1
P0ADE6	Uncharacteriz	0	8.05%	1	1	1
Q46908	Putative elect	2.18	6.56%	1	1	1
P0AGF2	Sulfur accepto	1.63	4.76%	1	1	1
Q46820	Uncharacteriz	0	1.72%	1	1	1
P42640	Putative phos	0	3.70%	1	1	1
P37624	Ribosome-ass	2.02	1.98%	1	1	1
P0ADN2	UPF0438 prot	1.62	14.29%	1	1	1
P0ADP2	Uncharacteriz	1.72	6.45%	1	1	1
P0ADP7	Ubiquinone bi	0	3.98%	1	1	1

P0A9V8	3-sulfolactald	1.61	3.69%	1	1	1
P68206	UPF0337 prot	2.02	17.39%	1	1	1
P39336	Uncharacteriz	2	1.99%	1	1	1
P39359	Probable 2-de	1.6	2.99%	1	1	1
P39367	Uncharacteriz	0	6.05%	1	1	1
P39368	Uncharacteriz	2.27	12.71%	1	1	1
P76146	UPF0187 prot	3.37	3.95%	1	1	1
P76173	Anaerobic din	2.15	5.63%	1	1	1
P76280	Uncharacteriz	0	10.09%	1	1	1
P76559	Uncharacteriz	1.71	6.63%	1	1	1

# PSMs	# AAs	MW [kDa]	calc. pI
22	468	51.4	5.15
10	396	43.5	5.77
2	319	35.2	6.04
2	449	49.3	7.11
8	304	33.3	7.68
1	434	47.5	5.31
1	1520	167.1	5.03
2	400	43.3	6.28
5	891	97.6	5.88
15	865	93.4	5.4
1	193	22.9	6.34
2	78	8.6	4.06
12	397	42.2	7.99
6	215	24.8	5.88
7	652	72	5.8
12	891	96.1	6.79
5	187	20.7	5.17
12	820	89.1	5.68
4	810	88.8	5.59
1	405	45.5	6.25
3	412	46.2	7.87
3	479	52.2	5.15
4	359	39.1	5.86
2	503	54.8	7.23
14	870	98.9	5.31
2	441	49.8	5.39
3	292	33.4	6.95
1	443	49.2	6.54
1	257	27	5.62
3	383	42.3	5.9
2	156	17	5.03
8	457	50.3	5.27
26	660	74.2	6.87
8	379	41.6	6.89
3	322	36.3	7.3
6	296	33.1	7.58
1	362	38.9	6.05
1	554	62.6	5.87
4	152	16.9	6.79
8	478	52.3	5.29
2	447	49.3	6.13
1	492	53	6.01
2	186	21.9	6.68
10	513	55.2	6.13
17	460	50.3	5.01
1	177	19.3	5.02
6	156	17.3	6.24
1	287	31.6	8.76
1	417	46.7	5.96
10	392	41.9	4.91

10	344	36.8	5.57
8	245	27.8	6.6
1	222	25	6.01
2	363	41	6.4
1	523	59.4	6.6
24	1024	116.4	5.5
2	479	55.3	5.86
3	220	25.1	6.65
17	883	99	5.68
2	382	41.4	6.38
22	1073	117.8	5.34
1	753	84.1	5.88
9	382	43.9	6.05
15	548	57.3	4.94
1	400	44.2	5.68
38	427	48	6.68
1	758	84.2	6.32
12	857	95.5	5.52
3	424	46.3	5.35
1	323	37	6.61
2	316	36.6	4.73
7	334	38	6.96
9	210	23.6	8.25
3	1047	114.6	6.48
2	365	41	7.5
7	324	36.1	7.42
1	201	22.3	6.37
6	273	29.3	6.52
3	570	64	7.64
3	323	34.5	6.06
6	475	52.5	5.14
5	403	44.4	8.28
6	432	47.6	6.62
3	428	46.1	5.81
2	273	28.7	5.76
1	274	29.9	5.74
4	375	41.2	5.52
3	193	21.2	5.91
5	681	77.5	5.74
6	185	19.8	5.33
1	629	70.5	8.72
16	490	55	5.85
2	472	51.4	4.73
5	367	40	5.58
8	447	48.6	6.4
1	434	47.3	8.85
13	588	64.4	6.27
9	238	26.8	6.73
1	716	81.4	7.71
8	474	50.7	6.15
5	571	64.6	6.67

2	271	29.4	5.96
8	467	52.5	8.66
24	638	69.1	4.97
2	928	103.1	5.58
8	1160	129.8	5.31
2	327	35.8	6.62
1	620	67.6	6.62
1	48	4.8	8.21
4	423	46.7	6.92
22	704	77.5	5.38
1	283	30.4	5.29
29	394	43.3	5.45
6	450	50.3	6.81
1	342	38.7	6.6
2	258	29.8	5.63
2	332	36.8	5.99
6	172	19	6.57
1	406	42.6	5.54
1	413	43	6.09
1	244	25.5	7.42
1	317	33.5	5.25
4	262	27.8	5.87
1	215	24.4	9.35
1	151	17	7.39
1	446	48.5	5.3
2	239	27	7.02
4	309	34.7	5.12
17	1016	112.5	7.28
5	300	33.1	5.33
1	260	27.7	4.81
1	192	21.6	8.12
4	288	31	6.09
6	414	47	6.95
1	167	18.9	5.31
2	420	45.3	6.24
1	103	11.6	7.25
6	644	70.7	6.24
10	497	54.5	4.5
6	591	64.9	5.9
1	382	40.5	5.27
8	548	60.3	6.55
1	467	50.5	6.58
3	331	35.5	7.11
9	491	55.7	5.76
6	549	61.5	6.29
2	482	51.7	5.6
3	426	45.7	6.16
8	346	37.4	6.38
6	284	30.8	6.34
26	420	47.1	5.77
3	222	24.8	7.33

19	957	104.3	6
3	312	35.3	6.8
4	477	52.8	6.62
5	728	84.3	6.37
7	431	48.7	6.14
1	657	73.5	6.06
3	321	34.7	6.52
3	445	47.5	6.02
16	609	66.9	5.87
16	469	51.9	5.47
2	542	58.9	6.64
4	396	44.1	8.43
16	501	56.7	7.44
17	502	56.2	5.5
1	475	53.3	7.78
1	115	12.9	4.75
5	417	45.3	6.48
3	192	20.8	6.42
1	252	27.4	4.72
3	215	24.1	5.66
2	494	54.8	6.37
2	426	45.3	4.84
1	518	58.2	5.39
2	619	70.5	5.29
4	201	22.9	6.21
8	525	58.6	5.39
8	875	96.9	5.2
10	804	89.9	6.06
1	296	32.8	6.42
1	684	77.9	8.27
2	181	21.2	9.67
2	320	35.9	6.65
1	457	52.7	6.06
6	334	37.6	6.68
3	213	22.9	9.25
5	419	45.5	6.43
11	426	48.3	5.99
5	299	33.3	5.63
7	355	40.3	6.18
5	356	39.3	5.08
1	257	28.6	8.16
1	137	15.5	5.47
1	334	36.9	7.05
1	1300	148.9	7.9
2	140	15.2	7.24
10	292	32.5	4.49
1	443	49.6	5.35
19	624	71.4	5.21
3	137	15.8	5.83
5	416	45.7	5.26
3	726	78.9	5.87

1	180	20.6	9.52
2	514	56.2	5.88
3	562	60.4	5.5
3	491	54	5.31
3	616	65.5	5.9
1	309	34.1	5.82
1	434	48.4	5.78
2	370	40.9	8.53
1	107	11.5	4.88
5	404	45.1	6.37
2	323	35.2	4.81
1	312	33.7	5.54
4	726	80	5.31
3	263	30	5.58
4	315	34.2	5.44
5	360	38.6	6.89
3	446	49.9	4.98
2	228	25.2	5.95
3	561	62.3	6.68
1	304	33.5	6.58
2	329	36.5	5.44
15	599	66.5	5.59
1	324	35.9	7.33
2	363	39.5	5.29
5	396	42.7	6.81
1	252	26.8	6.13
5	586	65.8	4.78
7	678	72.8	5.41
1	213	22.5	6.93
2	78	8.3	9.25
1	312	34.6	5.39
1	333	36.5	6.19
6	371	41	6.7
5	901	103.1	6.49
2	565	63.2	5.31
12	759	82.4	5.5
1	533	60.2	5.59
2	263	30.4	4.77
5	567	62.5	6.28
3	312	32.3	5.77
2	753	84.6	5.92
26	1227	135.9	5.07
1	343	37.8	6.38
6	271	29.4	5.29
10	1148	129.9	6.16
1	277	31.3	8.76
4	474	53.6	5.34
1	270	29.6	5.38
1	248	27.8	5.85
3	251	28	5.05
3	406	44.3	6.23

2	629	69.5	6.64
1	411	44	5.14
1	457	49.8	5.88
2	176	20.6	6.21
2	265	30.3	4.73
1	372	40.9	5.36
13	1486	170.1	5.35
3	234	27	5
7	440	50.5	4.89
3	419	44.8	6.16
1	495	53.3	5.73
1	355	37.8	9.73
5	853	95.2	5.55
1	410	47.3	5.63
7	335	37.8	4.97
2	379	40.1	9.51
3	236	25.8	5.1
3	294	33.6	4.74
1	504	55.2	5.94
1	469	52.2	6.49
1	153	17.4	5.14
4	220	25	5.74
25	596	68.2	6.42
10	445	49.3	6.86
12	908	100.2	6.25
6	180	20.5	5.48
21	495	54.8	4.64
1	390	43.3	4.88
22	933	105	6.49
9	405	44	5.81
29	887	99.6	5.68
50	630	66.1	5.17
7	346	37.2	6.42
5	239	27.3	6.39
14	680	77.1	5.26
4	337	37.2	6.1
6	334	37.2	7.75
3	112	12	7.87
1	269	28.1	5.9
7	173	18.8	6.8
2	283	31.6	6.28
4	752	83.8	6.68
8	630	70.2	5.66
2	540	59.6	5.71
2	427	46.2	5.92
10	485	52.9	5.39
1	443	50.1	5.99
20	760	85.3	6.01
3	546	58.3	5.71
2	386	43.1	6.68
2	346	39	5.97

12	241	27.4	5.31
2	815	93.1	5.9
17	797	90.5	7.39
5	711	77.1	5.21
2	378	43	5.3
2	164	18.1	5.8
26	623	68.1	5.07
16	688	80.4	8.92
19	792	87.4	5.06
4	513	58.1	7.14
1	293	31.9	4.48
1	417	44.6	5.64
4	367	39	6.54
1	232	25.9	9.64
1	451	52.8	8.98
6	748	83.7	5.78
11	575	63.5	4.87
5	714	77.1	5.41
8	150	16.9	5.29
1	94	10.2	6.27
8	505	56.5	5.52
4	212	23.2	5.87
15	1295	141.3	5.41
1	345	36.8	4.96
5	237	27	5.16
4	456	51.5	6.01
8	432	47.3	5.49
3	341	38.2	6.74
4	392	42.4	5.71
1	280	31.9	7.01
16	1320	143.7	5.99
5	472	53.1	4.93
4	545	60.3	5.94
4	305	33.7	5.64
3	460	49.4	7.24
18	968	109.7	5.17
3	498	56.4	6.48
10	577	63.3	5.6
3	744	83.8	6.77
8	365	41.2	4.75
3	529	59.5	5.97
3	421	47.1	7.68
6	419	47	7.25
1	313	34.7	9.23
4	441	49.6	5.48
11	736	82.8	5.97
7	376	43.5	4.81
3	165	17.7	8.98
1	142	14.9	9.63
1	142	16	9.91
1	123	13.5	10.42

7	144	15	11.18
2	136	15.3	11.22
3	127	14.4	11.05
1	117	12.8	10.42
1	115	13.1	10.62
3	118	13.5	11.47
4	273	29.8	10.93
1	59	6.5	10.96
1	65	7.3	11.78
1	201	22.1	9.73
17	179	20.3	9.48
3	177	18.9	9.7
1	209	23.3	9.41
1	396	44.3	7.72
3	384	43.1	6.99
7	326	37.1	6.81
5	644	72.4	5.62
3	226	25.5	6.89
50	1061	118.1	5.62
2	238	25.3	5.71
2	813	92.1	8.62
1	337	36.2	5.8
3	477	54	4.75
3	296	32.3	6.54
6	329	36.5	5.06
93	1342	150.5	5.26
29	1407	155.1	7.08
17	613	70.2	4.79
4	330	37.9	4.94
4	161	17.3	4.2
1	129	13.8	11.33
3	124	13.8	11
4	118	13.1	10.78
1	101	11.6	11.17
1	84	9.7	9.6
3	75	9	10.59
5	557	61.1	4.98
7	241	26.7	7.14
3	233	26	10.27
6	206	23.5	10.05
1	167	17.6	10.11
4	131	15.2	5.39
1	130	14.1	9.42
3	130	14.8	10.95
4	429	48.3	7.52
1	404	45.9	6.06
5	239	26.7	5.66
1	455	48.7	5.8
7	901	102	5.6
4	155	17.3	4.37
14	410	44.1	6.35

4	322	35	5.72
3	362	39.8	5.59
5	269	30.8	4.56
2	198	22.8	8.72
1	155	15.6	9.31
1	193	21.3	5.95
2	658	73.9	4.94
3	702	79.3	8.73
7	453	49.8	9.51
1	165	18.3	4.55
1	1120	113.7	7.65
4	466	51.5	6.57
16	388	41.4	5.52
7	289	29.8	6.79
1	495	54.7	5.16
2	267	29.2	6.96
27	876	96	5.81
3	461	52.2	5.57
10	590	65.9	5.69
2	471	53.8	5.94
3	327	36.8	6.2
9	795	87.3	5.3
2	303	34.7	5.05
35	689	76.8	5.44
8	424	47	5.91
12	938	104.2	6.01
14	505	57.6	5.24
16	505	57.8	5.24
14	860	97.2	5.3
12	677	76.2	5.86
12	466	52.5	5.31
2	554	63.4	6.28
3	577	64.6	5.49
6	430	48.4	5.5
12	642	74	6.19
25	951	108.1	5.34
1	334	37.4	6.74
2	424	47.5	5.81
3	529	59.3	5.22
7	1170	134	5.94
16	317	35.2	5.21
1	89	9.7	6.09
2	171	18.4	5.22
2	341	37.2	6.38
3	482	54.9	6.61
1	281	30.8	4.69
4	432	48.2	4.88
8	663	72.2	5.67
2	421	43.1	9.07
1	875	99.1	6.02
1	246	27	6

1	397	43	6.09
3	270	30.4	8.53
2	349	39.1	6.57
1	607	67.3	5.33
2	373	42	6.01
2	513	57.6	5.78
3	264	30.5	6.02
1	391	42.9	7.15
3	388	43.6	6.46
2	208	22.5	5.47
2	144	16.1	5.29
7	635	72	5.5
5	940	103.8	6.64
13	673	76.2	5.24
1	720	81.9	6.24
1	257	29.3	5.14
2	483	55.9	6.49
2	246	27.9	9.55
2	326	36.4	5.59
4	298	33.8	9.39
3	298	34.2	8.81
1	255	28.6	7.96
1	246	28	9.06
1	243	27.8	5
1	192	20.9	8.63
2	152	17.4	6
1	269	29.4	8.82
1	155	17.5	4.3
2	427	46.1	6
4	332	36.4	5.6
4	361	38.9	6.43
3	223	25	6.52
4	530	59.8	5.08
5	586	65.6	4.5
6	184	20.7	4.27
1	350	39.8	6.47
3	373	42.6	4.83
2	363	39.6	4.96
4	132	14.2	7.88
1	222	24.4	8.32
4	653	72.7	7.17
1	313	34.3	9.55
1	94	10.4	8.68
10	502	54.7	5.17
17	1018	113.2	7.11
4	341	38.5	5.2
1	246	26.4	4.88
8	453	51.2	6.19
1	156	18	9.1
3	488	54	6.14
1	443	51.4	8.62

1	1653	181.5	5.43
1	293	33.2	6.77
14	454	50.9	6.48
1	72	7.9	4.83
1	95	10.9	9.17
1	386	45	4.77
3	433	48.4	6.13
2	284	32.5	7.24
2	335	37.1	6.47
3	132	15	5.88
3	240	26.8	5.66
2	637	71.8	5.73
8	773	85.1	6.3
1	540	61.1	6.54
6	236	25.6	6.55
2	344	40.5	9.31
1	548	61.5	7.99
1	230	26.1	6.15
4	235	26.7	6.54
3	169	19	6.83
2	199	22.7	5.39
1	329	37.1	6.35
3	196	22.6	4.53
4	191	21.9	5.08
2	117	12	5.78
1	318	35.6	4.92
1	225	25.3	4.91
4	555	62.4	5.6
3	287	31.4	5.9
3	393	41	5.87
1	318	35.9	8.22
6	191	20	8.98
2	184	20.2	5.55
1	1758	182.5	4.87
3	247	28.3	6.79
3	327	36.6	5.35
2	141	16.2	6.38
11	231	24.4	7.18
2876	1706	177.4	4.78
12	472	52.2	5.58
1	451	50.6	5.83
1	484	54	6.34
1	183	19.8	5.41
3	474	53.9	5.76
1	344	38.4	6.46
1	322	35.8	6.52
2	79	8.3	4.61
1	467	52	7.66
3	779	86	6
2	490	52.9	5.31
1	603	68.4	5.45

1	346	38.6	5.48
1	647	71.3	9.63
2	231	25.5	4.98
1	207	23.2	5.8
1	292	33.3	4.7
2	457	51.6	5.83
1	133	15.6	6.79
2	848	97.5	6.21
2	291	32.5	6.09
3	354	39.2	6.3
1	407	44.3	5.29
1	229	26.1	5.67
1	356	38.2	4.98
1	204	23.3	4.97
1	376	41.1	7.84
4	643	71.1	6.84
1	167	18.7	6.11
1	211	23.5	8.16
1	419	46.6	9.91
1	114	12.1	4.31
1	204	22.7	7.33
1	387	40.8	6.8
1	91	10.9	6.3
2	233	26.2	5.49
1	1329	146.6	5.02
1	348	39.6	6.47
1	190	20.8	5.68
1	560	58.9	9.25
1	890	102.3	6.7
1	358	40.8	5.6
1	623	69.1	7.49
1	246	27.8	6.43
2	393	42.9	4.82
1	88	10	6.6
1	301	34.4	8.95
1	396	45.6	9.52
1	387	45.1	9.74
1	375	42.8	10.05
1	411	47.5	9.89
1	320	34.8	5.72
2	398	43.1	5.97
1	284	30.8	6.8
1	289	32.3	6.67
1	466	49.9	6.32
1	1538	169.3	6.76
2	207	23.5	8.84
1	241	26.8	5.99
2	193	21.3	8.68
1	341	36	6.54
4	694	78.5	6.58
1	478	53	5.36

1	723	80.4	6.18
1	459	53.1	5.6
1	1037	111.4	5.47
1	365	40.4	9.03
1	347	36.9	5.26
2	582	64.4	8.54
1	540	60.3	6.34
1	828	93	8.03
1	217	23.9	6.23
2	847	93.1	6.15
2	189	21.4	5.36
1	377	41.4	6.44
1	166	18.6	5.68
1	511	57.9	7.21
1	143	15.1	5.86
1	474	53.6	6.86
1	269	29.7	6.52
2	310	33.9	9.94
1	378	41.3	6.7
1	472	53.6	7.21
1	500	54.8	7.24
1	322	35.9	5.73
1	325	37	5.73
2	277	31.2	6.43
1	113	12.6	5.83
1	831	92.1	5.31
1	495	53.4	6.06
2	356	39.4	5.24
1	251	29.3	9.42
1	360	40.5	5.26
1	265	30.9	9.92
2	311	33.8	4.97
1	234	24.7	9.64
1	70	7.9	9.32
1	209	22.2	9.91
1	362	37.5	5.73
1	489	55.3	5.87
1	289	33.1	7.17
1	284	32.4	5.94
1	89	10.3	10.4
1	71	8.5	11.15
1	347	36.7	5.43
1	364	41.1	6.35
1	437	46.7	8.9
1	268	29.3	5.83
2	107	12.9	9.89
1	264	30.4	5.39
1	178	19	5.58
1	160	18.3	9.89
1	428	47.3	6.98
2	572	63.7	5.24

1	256	26.9	5.58
1	325	35	4.72
1	428	47.1	5.4
1	698	80.3	7.27
2	865	97.3	8.46
1	342	37.8	9.03
1	475	50.4	5.44
1	247	28	8.6
1	565	63.6	5.9
1	314	35.1	5.97
1	260	29.6	7.09
1	497	55.6	5.5
1	422	47.6	8.88
1	213	24.3	6.87
1	316	35.7	5.31
2	372	43.2	8.92
1	376	42.2	6.35
1	216	24.8	9.33
1	274	29.9	8.31
1	181	20.9	5.11
1	304	33.8	7.4
1	266	30	9.6
2	271	31.8	6.13
1	406	47.8	7.81
1	218	23.9	5.24
1	753	81.5	5.62
1	741	81.9	9.2
1	561	60.3	8.34
1	158	17.7	4.34
1	171	19	6.54
1	337	38.1	8.63
1	330	38.3	9.35
1	615	67.8	8.48
1	418	42.8	4.54
1	648	71.6	8.62
1	334	35.6	5.33
2	826	92.2	6.01
1	239	26.6	9.72
1	199	22.7	5.78
1	179	20.2	6.62
1	237	27.3	6.54
1	469	54	8.59
1	149	16.1	6
1	259	28.7	6.9
1	147	15.9	6.55
1	639	69	6.37
1	541	60.7	7.18
1	911	100.7	6.16
1	112	13.1	6.55
1	155	17.2	6.95
1	201	22.1	6.07

1	298	31.1	6.29
1	69	8.3	5.55
1	604	69.5	6.14
1	301	32.7	5.49
1	248	27.3	5.01
1	181	19.9	6.35
3	304	34.8	7.83
1	284	30.5	9.22
1	218	24.4	8.7
1	347	38.7	7.25
